# First line drug treatment for hypertension and reductions in blood pressure according to age and ethnicity: cohort study in UK primary care

**DOI:** 10.1136/bmj.m4080

**Published:** 2020-11-18

**Authors:** Sarah-Jo Sinnott, Ian J Douglas, Liam Smeeth, Elizabeth Williamson, Laurie A Tomlinson

**Affiliations:** 1 Department of Non-Communicable Disease Epidemiology, London School of Hygiene and Tropical Medicine, London WC1E 7HT, UK; 2 Department of Medical Statistics, London School of Hygiene and Tropical Medicine, London, UK

## Abstract

**Objective:**

To study whether treatment recommendations based on age and ethnicity according to United Kingdom (UK) clinical guidelines for hypertension translate to blood pressure reductions in current routine clinical care.

**Design:**

Observational cohort study.

**Setting:**

UK primary care, from 1 January 2007 to 31 December 2017.

**Participants:**

New users of angiotensin converting enzyme inhibitors/angiotensin receptor blockers (ACEI/ARB), calcium channel blockers (CCB), and thiazides.

**Main outcome measures:**

Change in systolic blood pressure in new users of ACEI/ARB versus CCB, stratified by age (< *v* ≥55) and ethnicity (black *v* non-black), from baseline to 12, 26, and 52 week follow-up. Secondary analyses included comparisons of new users of CCB with those of thiazides. A negative outcome (herpes zoster) was used to detect residual confounding and a series of positive outcomes (expected drug effects) was used to determine whether the study design could identify expected associations.

**Results:**

During one year of follow-up, 87 440 new users of ACEI/ARB, 67 274 new users of CCB, and 22 040 new users of thiazides were included (median 4 (interquartile range 2-6) blood pressure measurements per user). For non-black people who did not have diabetes and who were younger than 55, CCB use was associated with a larger reduction in systolic blood pressure of 1.69 mm Hg (99% confidence interval −2.52 to −0.86) relative to ACEI/ARB use at 12 weeks, and a reduction of 0.40 mm Hg (−0.98 to 0.18) in those aged 55 and older. In subgroup analyses using six finer age categories of non-black people who did not have diabetes, CCB use versus ACEI/ARB use was associated with a larger reduction in systolic blood pressure only in people aged 75 and older. Among people who did not have diabetes, systolic blood pressure decreased more with CCB use than with ACEI/ARB use in black people (reduction difference 2.15 mm Hg (−6.17 to 1.87)); the corresponding reduction difference was 0.98 mm Hg (−1.49 to −0.47) in non-black people.

**Conclusions:**

Similar reductions in blood pressure were found to be associated with new use of CCB as with new use of ACEI/ARB in non-black people who did not have diabetes, both in those who were aged younger than 55 and those aged 55 and older. For black people without diabetes, CCB new use was associated with numerically greater reductions in blood pressure than ACEI/ARB compared with non-black people without diabetes, but the confidence intervals were overlapping for the two groups. These results suggest that the current UK algorithmic approach to first line antihypertensive treatment might not lead to greater reductions in blood pressure. Specific indications could be considered in treatment recommendations.

## Introduction

High blood pressure, or hypertension, affects more than one in four adults globally and is a major modifiable risk factor for morbidity and mortality.^1^ Internationally, guideline based approaches to pharmacotherapy for hypertension have been adopted to simplify clinical practice and improve blood pressure control.^2^ ^3^ ^4^ Although some evidence suggests that the effectiveness of drug treatment for hypertension does not differ across the general population,^5^ ^6^ guideline recommendations hinge on the understanding that the effect of these drugs differs among specific subpopulations.

In the United Kingdom (UK), National Institute for Health and Care Excellence (NICE) guidelines recommend angiotensin converting enzyme inhibitors and angiotensin receptor blockers (ACEI/ARB) as first line treatment for hypertension in people younger than 55, and calcium channel blockers (CCBs) for people without diabetes aged 55 and older, replacing CCB with thiazides for those with drug intolerance.^2^ The presence of an age based recommendation is unique among major international guidelines for hypertension treatment,^3^ ^4^ and is based on differences in the activity of the renin-angiotensin system with age.^7^ ^8^ ^9^ Since this threshold was introduced in the first iteration of NICE hypertension guidance in 2004, the evidence base for hypertension treatment in older age, including the use and safety of ACEI/ARB drugs in older populations has evolved.^10^ ^11^

Furthermore, in NICE guidelines, use of CCB or thiazides is recommended as first line treatment for black people of African or Caribbean ethnic origin (referred to in this article as black people to reflect diversity). The same drugs are recommended, after consideration of comorbidities, in international guidelines.^2^ ^3^ ^4^ The pathophysiology of hypertension in this population has been thought to differ importantly from people of white heritage; lower levels of renin result in a reduced response to hypertension drugs that block the renin-angiotensin system such as ACEI/ARB.^12^ However, treatment recommendations based on historical categorisations of ethnicity have recently been criticised, because ethnicity can be considered a social construct rather than a biological one, and the proportion of people with mixed ethnic heritage has increased.^13^

Contemporary routine care is characterised by an increasingly older, more ethnically diverse and multi-morbid population. For people initiating hypertension drugs, it is not known whether current age and ethnicity based treatment recommendations translate to greater blood pressure reductions in these settings.

The Quality and Outcomes Framework in the UK ensures that blood pressure is regularly measured and recorded in patients’ electronic health records in primary care.^14^ Along with complete information on drugs prescribed, these anonymised data are a rich and high quality resource for examining drug effectiveness.^15^ Therefore, framing our questions around the current NICE algorithm for drug treatment of hypertension, we sought to determine whether initiation of CCB compared with ACEI/ARB led to differences in blood pressure reduction across age and ethnicity groups.

## Methods

### Data

We conducted a cohort study using the Clinical Practice Research Database (CPRD-GOLD) linked to Hospital Episodes Statistics. CPRD-GOLD is a nationally representative repository of prospectively collected, anonymised electronic health records from primary care in the UK, which has been extensively validated.^16^ It holds data on demographic information, health related behaviours, test results including blood pressure measurements, diagnoses, and prescriptions for more than 11 million people.^16^ The Hospital Episodes Statistics database records all hospital admissions for patients covered by the UK’s health service who receive treatment from either English NHS trusts or independent providers.^17^ Almost 60% of general practices included in CPRD-GOLD are linked to Hospital Episodes Statistics.^16^ We used linked data in this study to improve completeness of ethnicity recording.^18^

### Cohort entry

We identified new users of hypertension drugs (CCB, ACEI/ARB, and thiazides), defined as people who had a prescription for one of these drugs in the study period (1 January 2007 to 31 December 2017), with no previous use of these drugs in the preceding year. People entered the cohort (index date) on the date of the first prescription. People were eligible for inclusion from the latest of the following: study start date (1 January 2007), one year after general practice registration (to allow time for recording of covariates in the general practice record), the date when the general practices’ data recording processes were considered of adequate standard to be included in CPRD-GOLD, or the person’s 18th birthday. People remained in the cohort until the earliest of the following: end of the study period (31 December 2017), last data collection from the general practice, date of leaving the general practice, or death.

### Exclusion

We sought to study the association between first line drug treatment for hypertension and blood pressure solely in those individuals treated for hypertension. Therefore, we excluded people who initiated any of the study drugs without recorded blood pressure measurements in the year before cohort entry, and those whose blood pressure was at target or lower (<140/90 mm Hg, according to current NICE guidelines).^2^ We also excluded people who initiated more than one hypertension drug on the index date, as well as those with diabetes at baseline (because current NICE guidance recommends ACEI/ARB as first line hypertension treatment for all people with diabetes). Additionally, because ethnicity was a stratifying factor, we excluded people whose ethnicity was not determinable even after data linkage.

### Outcomes

Our main analysis looked at the change in systolic blood pressure from baseline at 12 weeks, 26 weeks, and 52 weeks, and repeated this for diastolic blood pressure in a secondary analysis. We included two types of control analyses: a positive outcome control and a negative outcome control.^19^ ^20^ The rationale of positive outcome analyses is that they demonstrate whether known drug-outcome associations can be replicated in a dataset. If such associations cannot be found, this could indicate confounding or problems with the data or methods. We included the following positive outcomes: incidence of ankle swelling, gout, and angioedema, all outcomes that we would expect to occur with different incidence according to drug prescribed. We also included a negative control outcome; herpes zoster. Our rationale in this instance was that none of the hypertension drugs studied should be causally associated with an altered risk of herpes zoster. If an association was found between any drug group and herpes zoster, this would suggest confounding or bias.^20^

### Covariates

Baseline values of systolic and diastolic blood pressure were taken from CPRD-GOLD, measured on or as close as possible to the index date. Using a priori knowledge about factors that could influence treatment choice or response we defined multiple covariates: age, sex, smoking, alcohol use, body mass index, diabetes, myocardial infarction, stroke, heart failure, arrhythmia, peripheral vascular disease, cancer, depression, and chronic obstructive pulmonary disease. Chronic comorbidities were defined using records from information recorded in both CPRD-GOLD and Hospital Episodes Statistics using all available data. We determined whether each person had ever previously been prescribed statins, antiplatelet agents, proton pump inhibitors, insulin, and loop diuretics. To capture polypharmacy, we measured drug treatment use from multiple British National Formulary chapters in the year before index date. To capture health service use in the year before index date, we determined how often a person visited their general practice. To account for socioeconomic status, we used person level deprivation based on the 2010 English Index Multiple Deprivation scores, separated into five equal groups, using quintiles. We calculated baseline estimated glomerular filtration rate from the most recent creatinine value recorded in CPRD-GOLD within one year before index date using the CKD-EPI (Chronic Kidney Disease Epidemiology Collaboration) equation. This variable was categorised into five groups: no CKD (estimated glomerular filtration rate ≥60), stage 3a (45-59), stage 3b (30-44), stage 4 (15-29), and stage 5 (<15).

To retain power in our main analysis, we imputed missing data under the missing-at-random assumption. Data for renal function were approximately 30% missing; our previous work has shown that people without recorded renal function have similar health outcomes to those with normal renal function after adjusting for covariates such as age, diabetes, and vascular disease.^21^ Data for other variables (smoking, alcohol, and body mass index) were approximately 5% missing. The imputation model included all explanatory variables listed above, including the outcome variable (first measurement of systolic and diastolic blood pressure during follow-up). We conducted diagnostics using the midiagplots function in Stata.^22^ We created five imputed datasets and combined treatment effects across these to obtain one overall estimate in each of our analyses.^23^

### Analysis

Reflecting current NICE guidance,^2^ we sought to answer two specific research questions in relation to blood pressure reduction. Firstly, in non-black people who do not have diabetes, is CCB versus ACEI/ARB for hypertension associated with different reductions in blood pressure in those younger than 55 versus those aged 55 and older? And secondly, in people who do not have diabetes, are CCB associated with different reductions in blood pressure than ACEI/ARB by black or non-black ethnicity? We also compared use of thiazides versus CCB to give insight into drugs that are recommended as alternative choices.^2^

Therefore, among new users of ACEI/ARB or CCB, within each of four groups defined by age (</≥55) and ethnicity (black/non-black), we estimated the propensity to be prescribed CCB versus ACEI/ARB using a logistic regression model including all the covariates listed above, except for diastolic blood pressure. Then, within each group, we matched one CCB user to one ACEI/ARB user on their propensity score within a caliper of 1%. We used linear mixed models to model change in systolic blood pressure with a random intercept for each person and fixed effects for exposure drug, using splines over time to allow the effects of exposure to vary flexibly over time. The fitted model was used to predict the blood pressure value at each follow-up (at 12 weeks, 26 weeks, and 52 weeks), so did not require patients to have measurements taken at those particular time points. In this way, the model could accommodate unbalanced data.^24^ Furthermore, this type of model can accommodate correlations within each person for longitudinal blood pressure measurements. The same analysis was used to compare thiazides versus CCB.

We used Cox proportional hazards models for the control outcomes within each of the propensity score matched groups defined above. For the gout and ankle swelling outcome models, we observed violation of the proportional hazards assumption. For these outcomes, we also modelled time specific hazard ratios. To reduce the risk of type I error with multiple testing, we defined a priori that we would report 99% confidence intervals.

### Subgroup and sensitivity analyses

To look more closely at CCB versus ACEI/ARB and thiazide versus CCB in reducing systolic blood pressure according to age in a non-black population that did not have diabetes, we used finer age categories for group specific analyses (age <55, 55 to <60, 60 to <65, 65 to <70, 70 to <75, and ≥75) and propensity score matched within each age group. We studied reductions in blood pressure associated with thiazide-like diuretics (indapamide and chlortalidone) versus CCB and also older thiazide agents versus CCB.

Our main analysis represented an intention-to-treat approach, so we also carried out an as-treated analysis whereby we censored follow-up blood pressure data if participants switched to or added another hypertension drug or discontinued their drug, defined as no repeat prescription within 90 days of previous prescription. This analysis aimed to examine the results for time spent on the drug initiated rather than the duration of follow-up that could include other drugs or no drugs. We carried out a complete case analysis to explore the impact of our multiple imputation approach. Finally, as a post hoc addition, we repeated our main analysis with 95% confidence intervals.

### Patient and public involvement

No patients were involved in setting the research question or the outcome measures, nor were they involved in developing plans for design or implementation of the study. No patients were asked to advise on interpretation or writing up of results.

## Results

**Fig 1:**
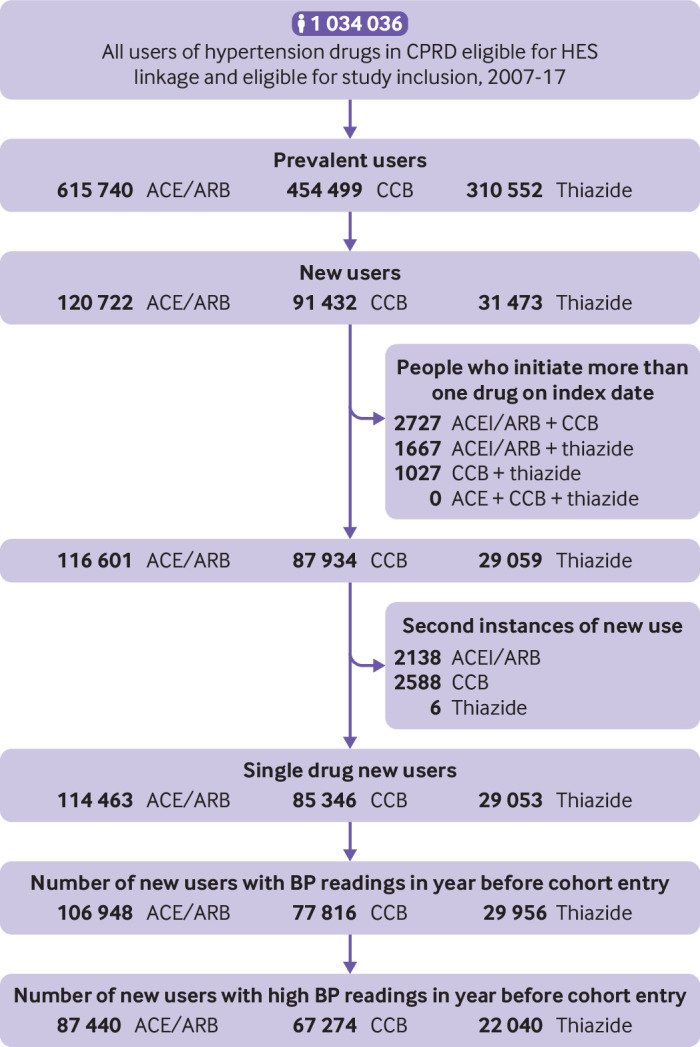
Study flowchart. CCB=calcium channel blockers; ACEI=angiotensin converting enzyme inhibitors; ARB=angiotensin receptor blockers; CPRD-GOLD=Clinical Practice Research Database; HES=Hospital Episodes Statistics; BP=blood pressure

From more than 1 million users of hypertension drugs between 2007 and 2017, we identified 87 440 new users of ACEI/ARB, 67 274 new users of CCB, and 22 040 new users of thiazides (fig 1). People taking thiazides were more likely to be female and were older than users of other hypertension drugs, while people taking ACEI/ARB group had a higher prevalence of myocardial infarction, heart failure, and diabetes (table 1). People without a blood pressure measurement during one year of follow-up were dropped from the blood pressure analysis (n=10 840). Demographics and comorbidity profiles were similar between those with and without blood pressure measurements (appendix 1).

**Table 1:** Descriptive characteristics for new users of angiotensin converting enzyme inhibitors/angiotensin receptor blockers (ACEI/ARB), calcium channel blockers (CCB), and thiazides at baseline. Data are number (%) of users unless stated otherwise

	ACEI/ARB (n=87 440)	CCB (n=67 274)	Thiazides (n=22 040)
**Sex**			
Female	37 158 (42.5)	33 506 (49.8)	14 277 (64.8)
**Age**			
<50	29 079 (33.3)	9204 (13.7)	2372 (10.8)
50-54	15 178 (17.4)	5377 (8.0)	1341 (6.1)
55-59	11 190 (12.8)	9522 (14.2)	2302 (10.4)
60-64	10 215 (11.7)	11 686 (17.4)	3394 (15.4)
65-69	7795 (8.9)	10 746 (16.0)	3205 (14.5)
70-74	5763 (6.6)	8421 (12.5)	3139 (14.2)
≥75	8220 (9.4)	12 318 (18.3)	6287 (28.5)
**Blood pressure (mm Hg; mean (standard deviation))**			
Systolic	160.2 (16.6)	165.8 (18.1)	164.3 (17.2)
Diastolic	94.8 (11.4)	93.8 (12.1)	91.5 (11.2)
**Ethnicity**			
White	73 247 (83.8)	55 477 (82.5)	19 253 (87.4)
South Asian	2949 (3.4)	1684 (2.5)	372 (1.7)
Black	914 (1.1)	3096 (4.6)	389 (1.8)
Other/mixed/unknown	3214 (3.7)	2394 (3.6)	707 (3.2)
Missing data	7116 (8.1)	4623 (6.9)	1319 (6.0)
**Index Multiple Deprivation scores (separated into five equal groups using quintiles)**			
Group 1 (least deprived)	20 174 (23.1)	15 540 (23.1)	40 491 (22.9)
Group 2	20 481 (23.4)	15 822 (23.5)	41 637 (23.6)
Group 3	17 616 (20.2)	13 457 (20.0)	35 634 (20.2)
Group 4	16 301 (18.6)	12 795 (19.0)	33 233 (18.8)
Group 5 (most deprived)	12 810 (14.7)	9,631 (14.3)	25 655 (14.5)
Missing data	58 (0.1)	29 (0.04)	17 (0.1)
**Body mass index**			
Underweight, <18.5	646 (0.7)	863 (1.3)	361 (1.6)
Healthy weight, 18.5-24.9	17 317 (19.8)	17609 (26.2)	6152 (27.9)
Overweight, 25-29.9	31 198 (35.7)	25 329 (37.7)	7755 (35.2)
Obesity, ≥30	35 119 (40.2)	20 498 (30.5)	6841 (31.0)
Missing data	3160 (3.6)	2975 (4.4)	931 (4.2)
**Smoking**			
Non-smoker	29 956 (34.3)	23 302 (34.6)	7984 (36.2)
Current smoker	18 183 (20.8)	12 632 (18.8)	3830 (17.4)
Ex-smoker	39 206 (44.8)	31 168 (46.3)	10 171 (46.1)
Missing data	95 (0.1)	172 (0.3)	55 (0.2)
**Alcohol**			
Non-drinker	7929 (9.1)	7060 (10.5)	2663 (12.1)
Current drinker	67 932 (77.7)	50 627 (75.3)	16 236 (73.7)
Ex-drinker	7332 (8.4)	6197 (9.2)	2093 (9.5)
Missing data	4247 (4.9)	3390 (5.0)	1048 (4.8)
**Comorbidities**			
Myocardial infarction	2489 (2.8)	449 (0.7)	158 (0.7)
Stroke	2671 (3.1)	2199 (3.3)	785 (3.6)
Heart failure	1122 (1.3)	294 (0.4)	114 (0.5)
Peripheral vascular disease	2427 (2.8)	2872 (4.3)	896 (4.1)
Diabetes	15 095 (17.3)	4289 (6.4)	975 (4.4)
Depression	6677 (7.6)	4756 (7.1)	1547 (7.0)
Chronic obstructive pulmonary disease	3141 (3.6)	3748 (5.6)	1250 (5.7)
Cancer	6455 (7.4)	7822 (11.6)	2729 (12.4)
Herpes zoster	4872 (5.6)	5067 (7.5)	1939 (8.8)
Gout	4365 (5.0)	3207 (4.8)	645 (2.9)
Angioedema	61 (0.1)	67 (0.1)	5 (0.0)
**Chronic kidney disease**			
No chronic kidney disease	57 963 (66.3)	37 990 (56.5)	11 824 (53.6)
Stage 3a	6132 (7.0)	4726 (7.0)	2126 (9.6)
Stage 3b	1541 (1.8)	999 (1.5)	393 (1.8)
Stage 4	218 (0.2)	251 (0.4)	44 (0.2)
Stage 5	28 (0.0)	96 (0.1)	—
Missing data	21 558 (24.7)	23 212 (34.5)	7651 (34.7)
**Drug treatments**			
Anti-platelet agents	11 868 (13.6)	9590 (14.3)	3699 (16.8)
Statins	19 769 (22.6)	13 740 (20.4)	4232 (19.2)
Proton pump inhibitors	29 775 (34.1)	26 374 (39.2)	8139 (36.9)
Insulin	2352 (2.7)	422 (0.6)	84 (0.4)
Loop diuretics	3109 (3.6)	2813 (4.2)	1607 (7.3)
Non-steroidal anti-inflammatory drugs	56 108 (64.2)	44 292 (65.8)	14 663 (66.5)
**No of primary care consultations**			
<5	22 829 (26.1)	17 330 (25.8)	5563 (25.2)
5-9	28 577 (32.7)	20 855 (31.0)	7237 (32.8)
10-14	16 106 (18.4)	12 243 (18.2)	4121 (18.7)
15-19	8091 (9.3)	6591 (9.8)	2081 (9.4)
≥20	11 837 (13.5)	10 255 (15.2)	3038 (13.8)
Missing data	0	0	0

For each drug comparison, we matched people on propensity score and achieved good balance on all covariates within age and ethnicity groups (appendices 2 and 3). Within the matched group of people with black ethnicity for each drug comparison, there was a 1 mm Hg difference in systolic blood pressure at baseline. A median of four (interquartile range 2-6) blood pressure measurements per person were available during the one year follow-up.

### Change in systolic blood pressure for CCB versus ACEI/ARB use by age

**Fig 2:**
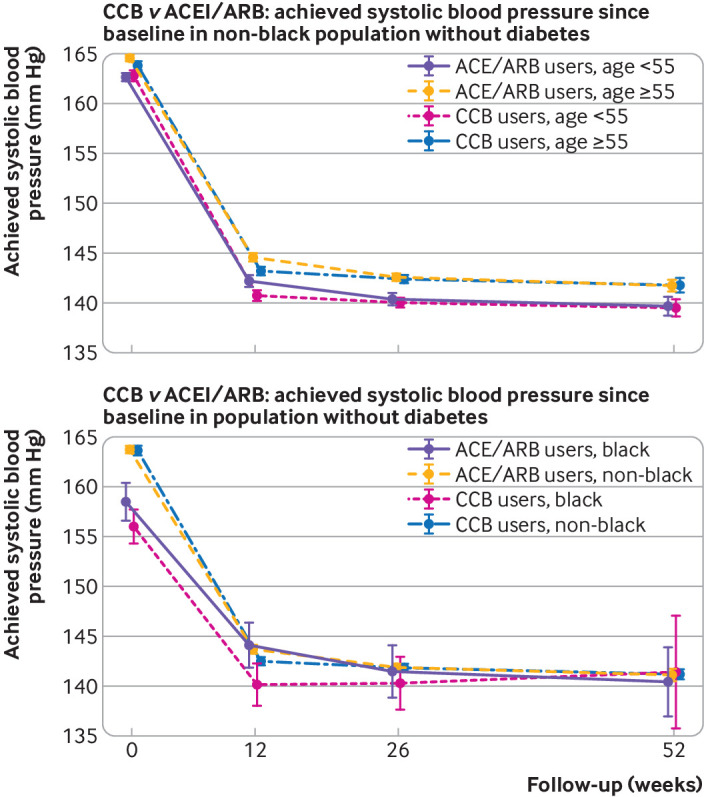
Achieved systolic blood pressures from baseline after new use of calcium channel blockers (CCB) versus new use of angiotensin converting enzyme inhibitors/angiotensin receptor blockers (ACEI/ARB) for hypertension in study groups, by study follow-up

In people younger than 55, systolic blood pressure changed from 162.8 mm Hg to 140.7 mm Hg at 12 weeks in new users of CCB, compared with a change from 162.7 mm Hg to 142.2 mm Hg in new users of ACEI/ARB. In relative terms, for people younger than 55, CCB use was associated with a reduction in systolic blood pressure of 1.69 mm Hg (99% confidence interval −2.52 to −0.86) more than ACEI/ARB use (table 2). In those aged 55 and older, systolic blood pressure changed from 163.8 mm Hg to 143.2 mm Hg at 12 weeks in new users of CCB, compared with a change from 164.6 mm Hg to 144.6 mm Hg in new users of ACEI/ARB (fig 2; table 2; relative difference −0.40 mm Hg (−0.98 to 0.18) in favour of CCB). Over one year’s follow-up, no difference was observed for reductions in systolic blood pressure associated with CCB and ACEI/ARB use in either age group (table 2 and fig 2).

**Table 2:** Difference in systolic blood pressure since initiation of hypertension drug treatment for study drug comparisons (CCB *v* ACEI/ARB; thiazide *v* CCB), by study group. Data are mm Hg (99% confidence interval)

Study group*	No	Follow-up period		
		12 weeks	26 weeks	52 weeks
**Difference in change of systolic blood pressure from baseline between CCB use and ACEI/ARB use†**				
Age <55	20 964	−1.69 (−2.52 to −0.86)	−0.48 (−1.47 to 0.51)	−0.53 (−1.96 to 0.91)
Age ≥55	58 396	−0.40 (−0.98 to 0.18)	0.63 (0.06 to 1.21)	0.85 (−0.11 to 1.81)
Non-black	73 726	−0.98 (−1.49 to −0.47)	0.11 (−0.42 to 0.64)	0.08 (−0.81 to 0.96)
Black	894	−2.15 (−6.17 to 1.87)	0.55 (−3.39 to 4.49)	2.28 (−5.36 to 9.92)
**Difference in change of systolic blood pressure from baseline between thiazide use and CCB use‡**				
Age <55	5724	1.51 (−0.03 to 3.06)	0.07 (−1.70 to 1.83)	0.34 (−2.39 to 3.07)
Age ≥55	32 464	2.16 (1.35 to 2.96)	1.43 (0.56 to 2.31)	0.17 (−0.95 to 1.30)
Non-black	35 876	2.10 (1.37 to 2.82)	1.19 (0.40 to 2.0)	0.32 (−0.74 to 1.38)
Black	648	−0.28 (−5.69 to 5.14)	−1.75 (−6.98 to 3.48)	2.01 (−5.46 to 9.47)

CCB=calcium channel blockers; ACEI=angiotensin converting enzyme inhibitors; ARB=angiotensin receptor blockers. All analyses are 1:1 matched by propensity score within each group. The total number for each stratum consists of half CCB and half ACEI/ARB (or half thiazide and half CCB). Appendix 6 shows changes in systolic blood pressure with 95% confidence intervals.

* Groups did not include people with diabetes.

† For the CCB *v* ACEI/ARB comparison, a negative result means that CCB use resulted in larger reductions in systolic blood pressure than ACEI/ARB use; a positive result indicates that ACEI/ARB use resulted in larger reductions in systolic blood pressure than CCB use.

‡ For the thiazide *v* CCB comparison, a negative result means thiazide use resulted in larger reductions in systolic blood pressure than CCB use; a positive result indicates that CCB use produced larger reductions in systolic blood pressure than thiazide use.

### Change in systolic blood pressure for CCB versus ACEI/ARB use by ethnicity

Among black people, systolic blood pressure changed from 156.0 mm Hg to 140.1 mm Hg at 12 weeks in new users of CCB, compared with a change from 158.4 mm Hg to 144.1 mm Hg in new users of ACEI/ARB. In relative terms, for black people, CCB use was associated with a reduction in systolic blood pressure of 2.15 mm Hg (99% confidence interval −6.17 to 1.87) more than ACEI/ARB use (table 2). Among non-black people, systolic blood pressure changed from 163.6 mm Hg to 143.5 mm Hg at 12 weeks in new users of CCB compared with a change from 163.7 mm Hg to 143.8 mm Hg in new users of ACEI/ARB (fig 2; relative reduction of 0.98 mm Hg (99% confidence interval −1.49 to −0.47) among CCB initiators). Over one year’s follow-up, no difference in reductions of systolic blood pressure was seen between CCB and ACEI/ARB use for either non-black or black people (table 2 and fig 2). We found results of similar magnitude and direction for diastolic blood pressure (appendices 4 and 5).

### Change in systolic blood pressure for thiazide use versus CCB use by age and ethnicity

**Fig 3:**
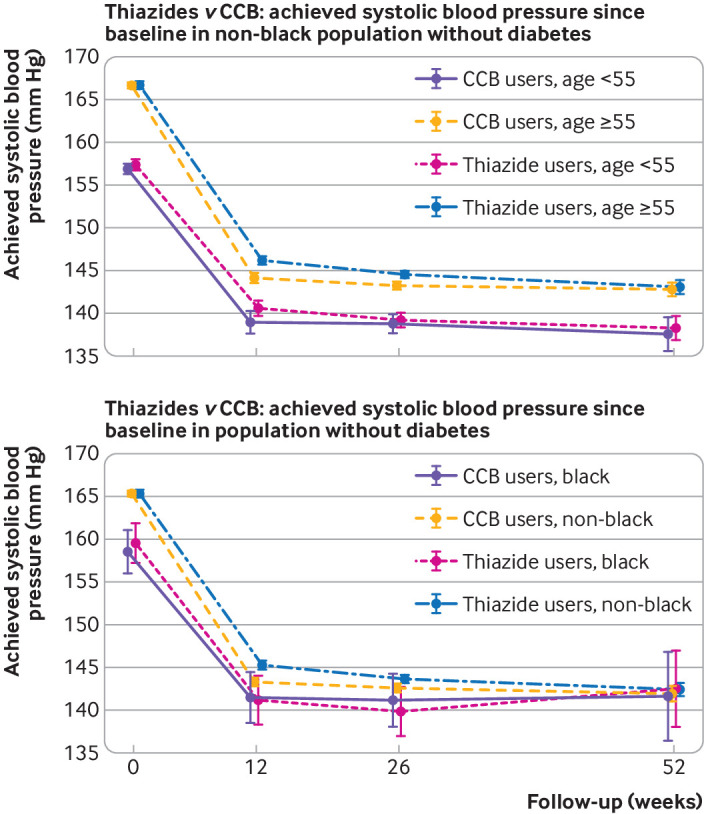
Achieved systolic blood pressures from baseline after new use of thiazide versus calcium channel blockers (CCB) for hypertension in study groups, by study follow-up

For people aged 55 and older, new use of CCB were associated with greater reductions in systolic blood pressure than new use of thiazides at 12 and 26 weeks. However, the reductions observed were similar to those for people younger than 55 years, indicated by overlapping confidence intervals. Similarly, overlapping confidence intervals for blood pressure reductions in each of the ethnicity groups indicated no evidence for a difference for thiazide versus CCB use in black and non-black people (table 2 and fig 3). We observed similar results for diastolic blood pressure (appendices 4 and 5).

### Control analyses

**Fig 4:**
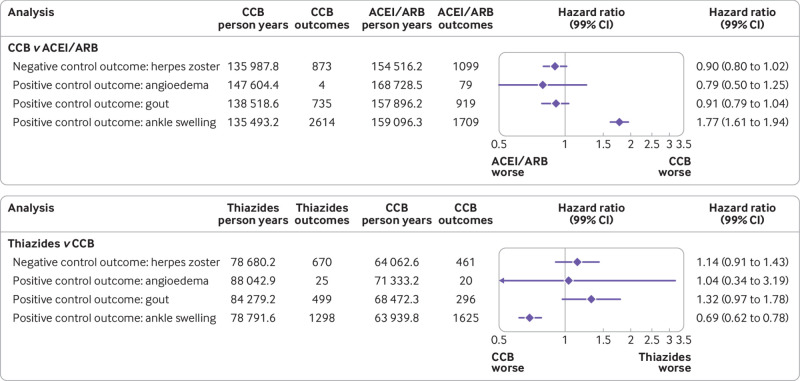
Positive and negative outcomes for entire study population (excluding people with diabetes) in hypertension drug comparison groups matched by propensity score. Positive and negative outcomes for each group level comparison are presented in appendix 7. CCB=calcium channel blockers; ACEI=angiotensin converting enzyme inhibitors; ARB=angiotensin receptor blockers

The incidence of drug specific side effects was as expected for each drug comparison; we found a higher incidence of ankle swelling for new users of CCB than for new users of ACEI/ARB (hazard ratio 1.77, 99% confidence interval 1.61 to 1.94), and a lower incidence of ankle swelling for new users of thiazides than for new users of CCB (0.69, 0.61 to 0.78; fig 4). We saw no evidence indicating that the incidence of herpes zoster differed between drug groups in either drug comparison (fig 4). Appendix 7 presents negative and positive control analyses as well as time specific hazard ratios for each study group.

### Sensitivity analyses

**Fig 5:**
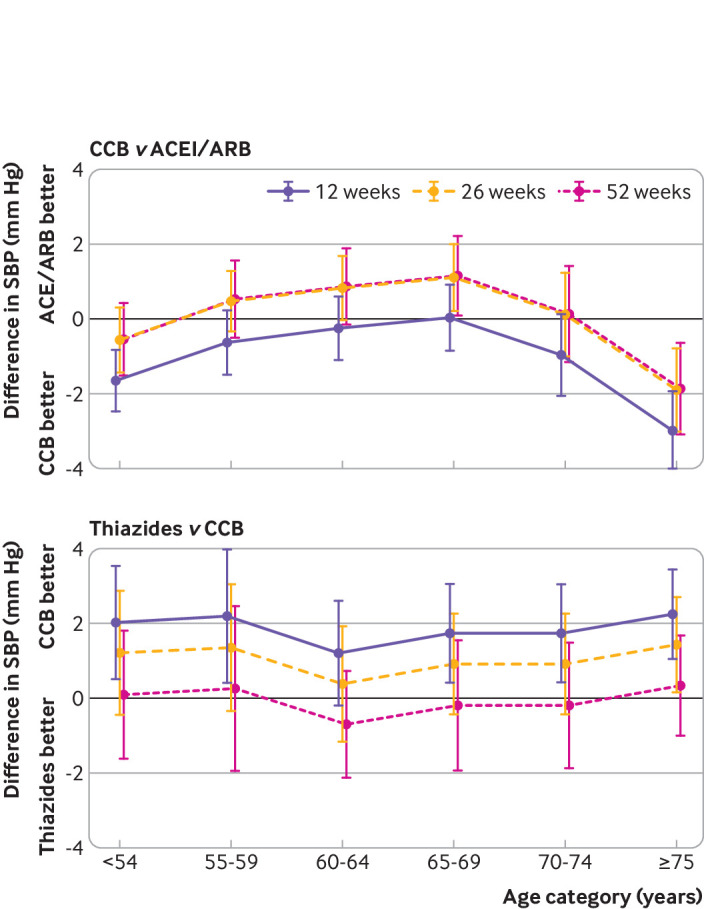
Difference in systolic blood pressure (SBP) across age groups for calcium channel blockers (CCB) versus angiotensin converting enzyme inhibitors/angiotensin receptor blockers (ACEI/ARB) and for thiazides versus CCB. Bars=99% confidence intervals

Across all age categories, among non-black people who did not have diabetes, we found that new use of CCB was associated with larger reductions in systolic blood pressure relative to new use of ACEI/ARB only in those aged 75 and older (12 week change −3.0 mm Hg (99% confidence interval −4.05 to −1.93), 26 weeks change −1.90 mm Hg (−3.03 to −0.77), 52 weeks change −1.86 mm Hg (−3.08 to −0.64); fig 5). CCB were associated with larger reductions than thiazides in all age categories at 12 weeks, but the difference between the drugs became negligible with increasing time (fig 5).

In the whole cohort matched by propensity score, we found that thiazide-like drugs (indapamide and chlortalidone) were of similar effectiveness to CCB in terms of lowering systolic blood pressure, and that CCB were more effective than older thiazide drugs (appendix 8). Our as-treated analysis provided results that were broadly similar to the main analysis. The numerical reduction in blood pressure was greater among black people commencing CCB than those commencing ACEI/ARB (−3.65 mm Hg, 99% confidence interval −7.99 to 0.70), compared with the corresponding numerical reduction observed in non-black people (−0.35 mm Hg, −0.94 to 0.24). However, the 99% confidence intervals were overlapping for both groups, indicating no strong evidence for a difference, similar to the main analysis (appendix 9). The complete case analysis found similar results to the main analysis (appendix 10).

## Discussion

### Principal findings

This propensity score matched cohort study included more than 150 000 people with hypertension in routine primary care in the UK. We found no evidence to support a difference in the blood pressure reductions observed for CCB versus ACEI/ARB among non-black people who did not have diabetes and who were aged above and below 55. In subgroup analyses, however, we found that CCB were associated with larger reductions in blood pressure than ACEI/ARB in people older than 75. Among people who did not have diabetes, our results suggested a numerically greater reduction in systolic blood pressure for CCB versus ACEI/ARB new use among black people compared with non-black people, but the confidence intervals for the reductions overlapped between the two groups indicating no strong evidence for a difference.

### Strengths and limitations

It is possible that people initiating hypertension drugs other than first line options suggested by current guidance were prescribed them for other indications. Despite good balance on baseline comorbidities in matched cohorts, residual confounding caused by unrecorded heart failure and kidney disease could have attenuated the blood pressure response to treatment. However, we endeavoured to reduce confounding by indication by including only people with hypertension; undertaking our analyses only in people who did not have diabetes; and matching by propensity score on multiple factors, inclusive of heart failure. This last approach achieved highly balanced cohorts for each drug comparison within each group. Furthermore, we used multiple strategies to help detect bias in our matched cohorts; our negative outcome analysis demonstrated no increased risk of herpes zoster in any drug group.^20^ Additionally, our positive outcome analyses showed that our data and methods were sensitive to drug effects anticipated to be causally associated with specific hypertension drugs. 

Our results related to ethnicity have wide margins of uncertainty, which could have occurred for several reasons. Firstly, we compared changes in blood pressure between black and non-black people, consistent with current UK guidance.^2^ Much of the original clinical research that informs this guidance compared people with an African or Caribbean ethnic origin versus people classified as white. If people of non-black and non-white ethnicity in our study had intermediate responses to the hypertension drug classes, their inclusion in the non-black group could have contributed to the overlapping confidence intervals observed for non-black and black people. Secondly, we had small numbers of black people initiating ACEI/ARB as a first line treatment for hypertension, suggesting that current NICE guidance is closely adhered to. Thirdly, the uncertainty we observed could have reflected the wide variation in the activity of the renin-angiotensin system across all ethnic groups.

Our outcomes relied on measurements of blood pressure at clinics because these measurements are recommended by NICE to monitor hypertension in primary care and to guide treatment decisions, except for patients suspected of white coat hypertension or resistant hypertension, in whom ambulatory or home monitoring is recommended.^2^ Therefore, the blood pressure measurements used in this study reflect the provision of care as it occurs routinely. Furthermore, we do not expect that any one method of blood pressure measurement was more or less concentrated in any one drug group, thus removing the risk of measurement bias. Our study did not look at the issue of adherence to hypertension drugs, although our as-treated analysis did censor individuals if they discontinued their index drug.

### Comparison with the literature and other studies

NICE guidelines recommend ACEI/ARB as the first line treatment for hypertension for those younger than 55.^2^ This age threshold, currently absent in international guidelines, is based on the idea that hypertension in younger people is more commonly characterised by high renin levels.^7^ ^8^ The age cut-off point in NICE guidelines is supported by studies limited by small sample size (<60 people)^7^ ^8^ and short follow-up periods (<6 weeks),^7^ ^8^ restricted to men^9^ and geographical location,^7^ and including unpublished data.^25^ To support drug recommendations in those aged 55 years and older, three trials are cited in the NICE guidance although none show clear effect modification by age (usually categorised as age <60/>60 or <65/>65) for any included clinical outcome.^26^ ^27^ ^28^ Our age stratified results are in line with a meta-analysis of 31 trials including more than 190 000 patients, which showed no difference in blood pressure reduction or cardiovascular events for any hypertension drug class between patients aged younger or older than 65.^29^ Our result of no difference in effectiveness between CCB and ACEI/ARB in new users aged 55 and older also accords with the results of a randomised study that examined effectiveness of first line treatment for lowering blood pressure between people aged younger than 55 and those aged 55 and older.^30^

Since first including 55 years as the age threshold in NICE guidance (CG18) in 2004, the literature has evolved to capture older populations with subgroup analyses based on older ages (comparison threshold at age 75) or subgroups defined by frailty.^10^ ^11^ In our subgroup analyses, greater reductions in blood pressure were found for CCB versus ACEI/ARB use only in those aged 75 and older. This finding could reflect an increased prevalence of isolated systolic hypertension in this subgroup (57% for age ≥75 *v* 40% for age ≥55 in our data), for which CCB might be more effective.^31^ ^32^ Although our results suggested no clear benefit in blood pressure reductions for CCB compared with ACEI/ARB in people aged 55 and older, the range of side effects might be as important as drug effectiveness in considering which hypertension drug class to prescribe. This decision is particularly important in older patients whose higher prevalence of comorbidities such as advanced chronic kidney disease could favour the use of CCB.

International guidelines for hypertension are similar to NICE guidelines in that ethnicity is a major consideration in the choice of a first line hypertension drug, although they do not use the algorithmic approach of UK guidance. Ethnicity based recommendations are supported by strong evidence from the ALLHAT randomised clinical trial, which showed use of thiazides or CCB was more effective than ACEI/ARB at lowering blood pressure in black people.^28^ We have suggested above several reasons why our results might differ from established clinical evidence. However, the value of making clinical decisions based on ethnicity is increasingly questioned given the growing proportion of people who classify themselves as multiethnic or of mixed heritage.^13^ Furthermore, our understanding of interactions between genetic and environmental factors in the development of hypertension is improving.^33^ Thus, the value of using categorisations of ethnicity as a proxy predictor for drug response is uncertain in current society, although is likely to be informed by ongoing research.^34^

### Conclusion

In conclusion, we observed reductions in blood pressure for CCB that were similar in magnitude to those for ACEI/ARB in people aged above and below 55. This finding suggests that some people aged 55 and older who are currently offered CCB, but have indications for renin-angiotensin blockade such as proteinuria, could be missing the therapeutic benefits of ACEI/ARB. We found no evidence that CCB new use was associated with larger reductions in blood pressure than ACEI/ARB new use among black people versus other ethnic groups, although power was limited and uncertainty existed in the estimate. Our results suggest that the algorithm for choice of pharmacotherapy in UK NICE guidance could be simplified. Moving towards a choice of any of the three major hypertension drug classes with suggested compelling indications for their use would align the UK with international guidance, in particular with regard to age.

**What is already known on this topic**

Current NICE recommendations for first line hypertension treatment are based on age, ethnicity, and diabetes
Excluding people with diabetes, calcium channel blockers (CCB) are recommended for those aged 55 and older and for people of black African or African-Caribbean family ethnicity (referred to here as black people to reflect diversity); angiotensin converting enzyme inhibitors or angiotensin receptor blockers (ACEI/ARB) are recommended for non-black people younger than 55
Since the current guidance was developed, the medical literature has evolved incrementally with more trials including older populations; meta-analysis of these new data indicate that the effectiveness of first line antihypertensive drugs does not differ by age (</>65); additionally, categorisations of ethnicity to guide clinical decision making have been questioned
It is not known whether recommended drug choices lead to greater reductions in blood pressure in current routine clinical care

**What this study adds**

In this propensity score matched cohort study excluding people with diabetes, initiation of either CCB or ACEI/ARB for hypertension was associated with similar reductions in blood pressure in people younger than 55 and those aged 55 and older; subgroup analyses indicated that CCB was associated with greater reductions in blood pressure than ACEI/ARB only in those aged 75 and older
Reductions in blood pressure appeared to be numerically greater for black people initiating CCB versus ACEI/ARB than those blood pressure reductions in non-black people, but the confidence intervals overlapped between the two groups
The finding that ACEI/ARB and CCB are associated with similar reductions in blood pressure in those aged above and below 55 suggests that age might not be the best factor to determine drug choice; other characteristics, such as level of urinary protein loss, could favour choice of a specific class of drug

## Web extra

Extra material supplied by authors

Web appendix: Appendices

## Notes

Contributors: S-JS, LAT, IJD, EW, and LS conceived and devised the study, S-JS and EW analysed the data. All authors contributed to the interpretation of the data. S-JS drafted the article and all authors (IJD, EW, LS, LAT) reviewed and edited the manuscript, and approved the version to be published. The corresponding author attests that all listed authors meet authorship criteria and that no others meeting the criteria have been omitted. S-JS is guarantor and accepts full responsibility for the conduct of the study, had access to the data, and controlled the decision to publish.

Ethical approval: The study protocol was approved by the London School of Hygiene and Tropical Medicine’s ethics committee (No 16251) and the Independent Scientific Advisory Committee for the Medicines and Healthcare products Regulatory Agency (No 18_312R). The protocol was made available to reviewers as supplementary material.

Data sharing: No additional data available.

The manuscript’s guarantor (SJS) affirms that the manuscript is an honest, accurate, and transparent account of the study being reported; that no important aspects of the study have been omitted; and that any discrepancies from the study as planned have been explained.

Dissemination to participants and related patient and public communities: We are not able to disseminate the results of the research directly to study participants because the data used were anonymised.

Provenance and peer review: Not commissioned; externally peer reviewed.
