# Supplementary material for: First line drug treatment for hypertension and reductions in blood pressure according to age and ethnicity: cohort study in UK primary care
Source: BMJ. 2020 Nov 18;371:m4080. doi: 10.1136/bmj.m4080 (PMC7670766; doi:10.1136/bmj.m4080)
Supplement: Supplementary file 1 — Web appendix: Appendices [file sins053556.ww.pdf]

List of Appendices

Appendix 1: Description of cohort meeting entry criteria, cohort with follow-up BP data and cohort without follow-up BP data 2

Appendix 2: Flowcharts, descriptive characteristics and propensity score distributions before and after matching, CCB vs ACE-I/ARB 3

Appendix 3: Descriptive characteristics and propensity score distributions before and after matching, thiazides vs CCB 7

Appendix 4: Diastolic blood pressure results........ 11

Appendix 5: Diagrams of diastolic blood pressure results........ 12

Appendix 6: 95% CI for changes in systolic blood pressure........ 14

Appendix 7: Negative and positive outcomes 15

Appendix 8: Comparison of thiazide-like diuretics with calcium channel blockers........ 18

Appendix 9: On treatment analysis........ 19

Appendix 10: Complete case analysis........ 20

Appendix 11: RECORD-PE checklist........ 21

Appendix 1: Description of cohort meeting entry criteria, cohort with follow-up BP data and cohort without follow-up BP data

Appendix 1 Table 1: Participant demographics comparing those entering the cohort, those with and those without follow-up data, by initiated drug class

	**ACEI/ARB**			**CCB**			**Thiazide**		
	Cohort meeting entry criteria n= 87,440	Cohort with FU data n = 82,706	Cohort without FU data n = 4,734	Cohort meeting entry criteria n = 67,274	Cohort with FU data n = 62,803	Cohort without FU data n = 4,471	Cohort meeting entry criteria n=22,040	Cohort with FU data n = 20,405	Cohort without FU data n = 1,635
Female	37,158 (42.5)	35,517 (42.9)	1641 (34.7)	33,506 (49.8)	31,454 (50.1)	2,052 (45.9)	14,277 (64.8)	13,179 (64.6)	1,098 (67.2)
Age ≥55yrs	43,183 (49.4)	41,047 (49.6)	2,136 (45.1)	52,683 (78.3)	49,578 (78.9)	3,115 (69.7)	18,327 (83.2)	17,137 (84.0)	1,190 (72.8)
Ethnicity:									
White	43,391 (49.6)	41,168 (49.8)	12,223 (47.0)	33,143 (49.3)	31,048 (49.4)	2,095 (46.9)	10,166 (46.1)	9,458 (46.4)	708 (43.3)
South Asian	2,384 (2.7)	2,213 (2.7)	171 (3.6)	1,406 (2.1)	1,280 (2.0)	126 (2.8)	290 (1.3)	269 (1.3)	21 (1.3)
Black	706 (0.8)	640 (0.8)	66 (1.4)	2,550 (3.8)	2,313 (3.7)	237 (5.3)	297 (1.4)	269 (1.3)	28 (1.7)
Other/mixed	850 (1)	781 (0.9)	69 (1.5)	739 (1.1)	676 (1.1)	63 (1.4)	137 (0.6)	121 (0.6)	16 (1)
Missing	40,109 (45.9)	37,904 (45.8)	2,205 (46.6)	29,436 (43.8)	27,486 (43.8)	1,950 (43.6)	11,150 (50.6)	10,288 (50.4)	862 (52.7)
Diabetes	15,095 (17.3)	14,238 (17.2)	857 (18.1)	4,289 (6.4)	3,971 (6.3)	318 (7.1)	975 (4.4)	898 (4.4)	77 (4.7)
MI	2,489 (2.9)	2,283 (2.8)	206 (4.4)	449 (0.7)	401 (0.6)	48 (1.1)	158 (0.7)	147 (0.7)	11 (0.7)
Stroke	2,671 (3.1)	2,478 (3.0)	193(4.1)	2,199 (3.3)	2,000 (3.2)	199 (4.5)	785 (3.6)	727 (3.6)	58 (3.6)
Heart Failure	1,122 (1.3)	1,008 (1.2)	114 (2.4)	294 (0.4)	269 (0.4)	25 (0.6)	114 (0.5)	108 (0.5)	6(0.4)
Smoking (yes)	18,183 (20.8)	16,942 (20.5)	1,241 (26.2)	12,632 (18.8)	11,546 (18.4)	1,086 (24.3)	3,830 (17.4)	3,472 (17.2)	358 (21.9)

Appendix 2: Flowcharts, descriptive characteristics and propensity score distributions before and after matching, CCB vs ACE-I/ARB

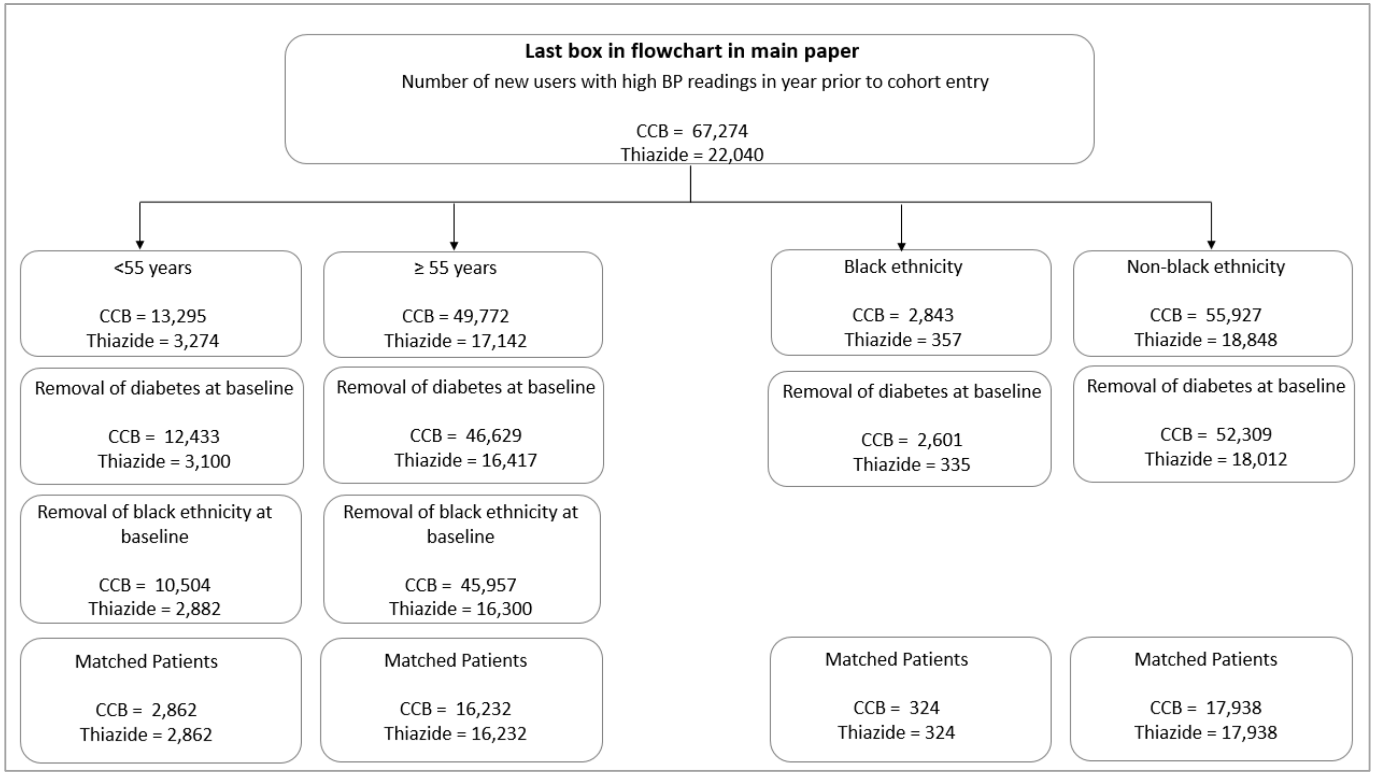

Appendix 2 Figure 1: Flowchart describing construction of each stratum for CCB versus ACEI/ARB comparisons

Appendix 2 Table 1 : Descriptive characteristics for initiators of calcium channel blockers (CCB) *versus* initiators of ACE-inhibitors/angiotensin receptor blockers (ACE-I/ARB) in matched stratum specific cohorts

	**Aged <55yrs**		**Aged ≥55yrs**		**Non-Black**		**Black**	
	ACE-I/ARB	CCB	ACE-I/ARB	CCB	ACE-I/ARB	CCB	ACE-I/ARB	CCB
	10482	10482	29198	29198	36863	36863	447	447
Females	4620 (44.1)	4675 (44.6)	13768 (47.2)	13549 (46.4)	17249 (46.8)	17380 (47.1)	251 (56.2)	250 (55.9)
Age (years)								
<50	6256 (59.7)	6278 (59.9)	0 (0.0)	0 (0.0)	5723 (15.5)	5723 (15.5)	258 (57.7)	258 (57.7)
50-54	4226 (40.3)	4204 (40.1)	0 (0.0)	0 (0.0)	3801 (10.3)	3801 (10.3)	75 (16.8)	75 (16.8)
55-59	0 (0.0)	0 (0.0)	7403 (25.4)	7403 (25.4)	6669 (18.1)	6669 (18.1)	42 (9.4)	42 (9.4)
60-64	0 (0.0)	0 (0.0)	7229 (24.8)	7229 (24.8)	6591 (17.9)	6591 (17.9)	22 (4.9)	22 (4.9)
65-69	0 (0.0)	0 (0.0)	5417 (18.6)	5417 (18.6)	5055 (13.7)	5055 (13.7)	20 (4.5)	20 (4.5)
70-74	0 (0.0)	0 (0.0)	3818 (13.1)	3818 (13.1)	3646 (9.9)	3646 (9.9)	19 (4.3)	19 (4.3)
75+	0 (0.0)	0 (0.0)	5331 (18.3)	5331 (18.3)	5378 (14.6)	5378 (14.6)	11 (2.5)	11 (2.5)
Ethnicity**								
White	8627 (82.3)	8247 (78.7)	25797 (88.4)	25593 (87.7)	34686 (94.1)	34172 (92.7)	0 (0.0)	0 (0.0)
South asian	415 (4.0)	588 (5.6)	442 (1.5)	515 (1.8)	827 (2.2)	1077 (2.9)	0 (0.0)	0 (0.0)
Black	0 (0.0)	0 (0.0)	0 (0.0)	0 (0.0)	0 (0.0)	0 (0.0)	447 (100.0)	447 (100.0)
Other/mixed/unknown	421 (4.0)	673 (6.4)	893 (3.1)	925 (3.2)	1350 (3.7)	1614 (4.4)	0 (0.0)	0 (0.0)
Missing	1019 (9.72)	974 (9.29)	2066 (7.08)	2165 (7.41)	0 (0.00)	0 (0.00)	0 (0.00)	0 (0.00)
BMI**								
Underweight <18.5	71 (0.7)	67 (0.6)	348 (1.2)	323 (1.1)	386 (1.0)	378 (1.0)	0 (0.0)	2 (0.4)
Healthy weight 18.5-24.9	1893 (18.1)	1857 (17.7)	7863 (26.9)	7758 (26.6)	8969 (24.3)	8975 (24.3)	77 (17.2)	81 (18.1)
Overweight 25-29.9	3402 (32.5)	3438 (32.8)	11505 (39.4)	11653 (39.9)	13976 (37.9)	13917 (37.8)	178 (39.8)	179 (40.0)
Obesity >=30	4718 (45.0)	4663 (44.5)	8260 (28.3)	8267 (28.3)	12119 (32.9)	12093 (32.8)	178 (39.8)	178 (39.8)
Missing	398 (3.8)	457 (4.4)	1222 (4.2)	1197 (4.1)	1413 (3.8)	1500 (4.1)	14 (3.1)	7 (1.6)
SMOKING**								
Non-smoker	3874 (37.0)	3835 (36.6)	9774 (33.5)	9785 (33.5)	12379 (33.6)	12557 (34.1)	250 (55.9)	246 (55.0)
Current smoker	2740 (26.1)	2756 (26.3)	4931 (16.9)	4823 (16.5)	7122 (19.3)	7087 (19.2)	64 (14.3)	58 (13.0)
Ex-smoker	3859 (36.8)	3878 (37.0)	14458 (49.5)	14511 (49.7)	17328 (47.0)	17136 (46.5)	133 (29.8)	143 (32.0)
Missing	9 (0.1)	13 (0.1)	35 (0.1)	79 (0.3)	34 (0.1)	83 (0.2)	0 (0.0)	0 (0.0)
ALCOHOL**								
Non-drinker	1055 (10.1)	1028 (9.8)	2499 (8.6)	2475 (8.5)	3202 (8.7)	3129 (8.5)	124 (27.7)	120 (26.8)
Current drinker	8094 (77.2)	8086 (77.1)	22763 (78.0)	23019 (78.8)	28856 (78.3)	28885 (78.4)	264 (59.1)	262 (58.6)
Ex-drinker	822 (7.8)	799 (7.6)	2391 (8.2)	2362 (8.1)	2978 (8.1)	3099 (8.4)	38 (8.5)	48 (10.7)
Missing	511 (4.9)	569 (5.4)	1545 (5.3)	1342 (4.6)	1827 (5.0)	1750 (4.7)	21 (4.7)	17 (3.8)
Comorbidities								
Myocardial Infarction	14 (0.1)	16 (0.2)	326 (1.1)	335 (1.1)	371 (1.0)	347 (0.9)	1 (0.2)	0 (0.0)
Stroke	157 (1.5)	159 (1.5)	1225 (4.2)	1325 (4.5)	1438 (3.9)	1461 (4.0)	6 (1.3)	8 (1.8)
Heart Failure	11 (0.1)	11 (0.1)	205 (0.7)	215 (0.7)	267 (0.7)	225 (0.6)	2 (0.4)	3 (0.7)
Peripheral Vascular Disease	119 (1.1)	121 (1.2)	1159 (4.0)	1161 (4.0)	1279 (3.5)	1208 (3.3)	2 (0.4)	6 (1.3)
Diabetes	0 (0.0)	0 (0.0)	0 (0.0)	0 (0.0)	0 (0.0)	0 (0.0)	0 (0.0)	0 (0.0)
depression	1022 (9.8)	994 (9.5)	1746 (6.0)	1684 (5.8)	2658 (7.2)	2674 (7.3)	23 (5.1)	34 (7.6)
COPD	168 (1.6)	170 (1.6)	1645 (5.6)	1594 (5.5)	1812 (4.9)	1729 (4.7)	5 (1.1)	8 (1.8)
Cancer	380 (3.6)	376 (3.6)	3581 (12.3)	3578 (12.3)	3889 (10.5)	3819 (10.4)	14 (3.1)	21 (4.7)
Herpes Zoster	391 (3.7)	359 (3.4)	2312 (7.9)	2345 (8.0)	2553 (6.9)	2575 (7.0)	10 (2.2)	10 (2.2)
Gout	407 (3.9)	409 (3.9)	1555 (5.3)	1587 (5.4)	1864 (5.1)	1882 (5.1)	11 (2.5)	9 (2.0)
Angioedema	16 (0.2)	17 (0.2)	15 (0.1)	11 (0.0)	25 (0.1)	27 (0.1)	0 (0.0)	1 (0.2)
CKD**								
no CKD	7041 (67.2)	5619 (53.6)	17340 (59.4)	15650 (53.6)	22626 (61.4)	19675 (53.4)	271 (60.6)	238 (53.2)
stage 3a	156 (1.5)	139 (1.3)	3556 (12.2)	3166 (10.8)	3550 (9.6)	3260 (8.8)	30 (6.7)	31 (6.9)
stage 3b	32 (0.3)	35 (0.3)	784 (2.7)	681 (2.3)	852 (2.3)	724 (2.0)	4 (0.9)	5 (1.1)
stage 4	15 (0.1)	9 (0.1)	121 (0.4)	93 (0.3)	131 (0.4)	112 (0.3)	2 (0.4)	2 (0.4)
stage 5	5 (0.0)	14 (0.1)	13 (0.0)	33 (0.1)	13 (0.0)	45 (0.1)	1 (0.2)	1 (0.2)
Missing	3233 (30.8)	4666 (44.5)	7384 (25.3)	9575 (32.8)	9691 (26.3)	13047 (35.4)	139 (31.1)	170 (38.0)
Drugs								

Antiplatelet agents	516 (4.9)	496 (4.7)	4625 (15.8)	4731 (16.2)	4996 (13.6)	5084 (13.8)	26 (5.8)	29 (6.5)
Statins	742 (7.1)	751 (7.2)	6456 (22.1)	6542 (22.4)	7038 (19.1)	6967 (18.9)	31 (6.9)	34 (7.6)
Proton Pump Inhibitors	3270 (31.2)	3213 (30.7)	11057 (37.9)	10853 (37.2)	13830 (37.5)	13754 (37.3)	149 (33.3)	156 (34.9)
Insulin	0 (0.0)	0 (0.0)	0 (0.0)	0 (0.0)	0 (0.0)	0 (0.0)	0 (0.0)	0 (0.0)
Loop diuretics	216 (2.1)	194 (1.9)	1331 (4.6)	1332 (4.6)	1481 (4.0)	1584 (4.3)	9 (2.0)	12 (2.7)
NSAIDs	6478 (61.8)	6537 (62.4)	19290 (66.1)	19380 (66.4)	24392 (66.2)	24547 (66.6)	286 (64.0)	303 (67.8)
Primary Care Consultations**								
<5 consultations	3351 (32.0)	3370 (32.2)	7734 (26.5)	7794 (26.7)	9633 (26.1)	9639 (26.1)	128 (28.6)	130 (29.1)
5-9 consultations	3045 (29.0)	3055 (29.1)	9925 (34.0)	10039 (34.4)	12300 (33.4)	12284 (33.3)	139 (31.1)	117 (26.2)
10-14 consultations	1687 (16.1)	1654 (15.8)	5268 (18.0)	5269 (18.0)	6736 (18.3)	6834 (18.5)	75 (16.8)	87 (19.5)
15-19 consultations	800 (7.6)	823 (7.9)	2598 (8.9)	2521 (8.6)	3357 (9.1)	3308 (9.0)	49 (11.0)	57 (12.8)
>=20 consultations	1599 (15.3)	1580 (15.1)	3673 (12.6)	3575 (12.2)	4837 (13.1)	4798 (13.0)	56 (12.5)	56 (12.5)
Missing	0 (0.0)	0 (0.0)	0 (0.0)	0 (0.0)	0 (0.0)	0 (0.0)	0 (0.0)	0 (0.0)
Systolic BP mean (sd)	162.7 (17.2)	162.8 (19.2)	164.6 (16.8)	163.8 (16.2)	163.7 (17.0)	163.6 (17.1)	158.4 (15.4)	156.0 (14.5)
Diastolic BP mean (sd)	100.4 (10.0)	101.0 (11.3)	92.4 (11.2)	92.5 (11.2)	94.2 (11.5)	94.5 (11.9)	96.7 (10.2)	96.9 (9.9)

<table>
<tr><th></th><th>Before Matching</th><th>After Matching</th></tr>
<tr><td><55years</td><td>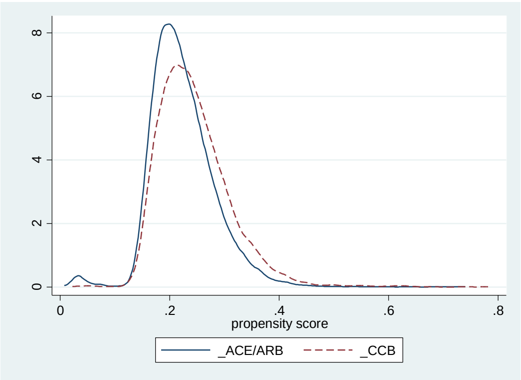
</td><td>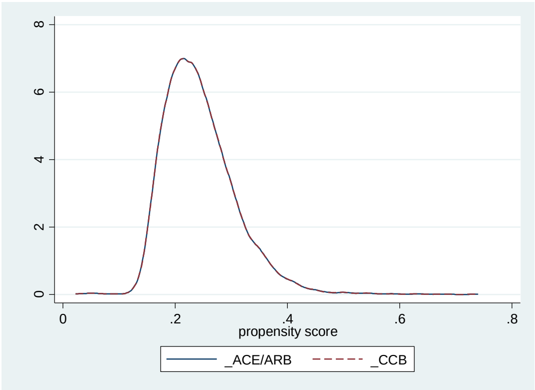
</td></tr>
<tr><td>≥55years</td><td>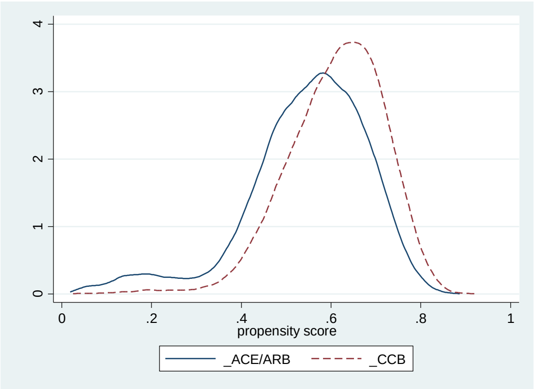
</td><td>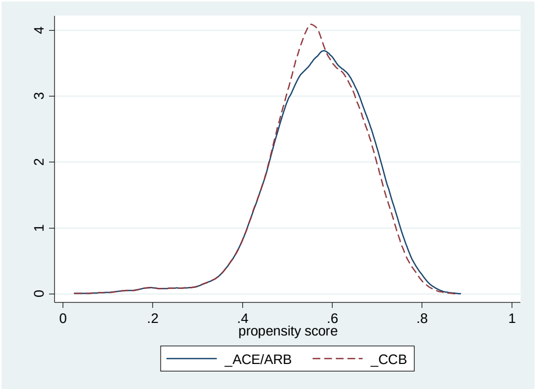
</td></tr>
<tr><td></td><td></td><td></td></tr>
<tr><td>Non-Black</td><td>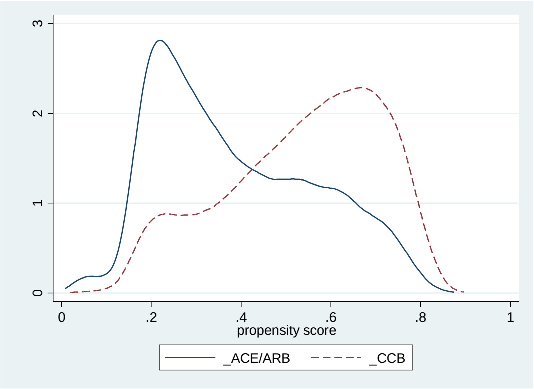
</td><td>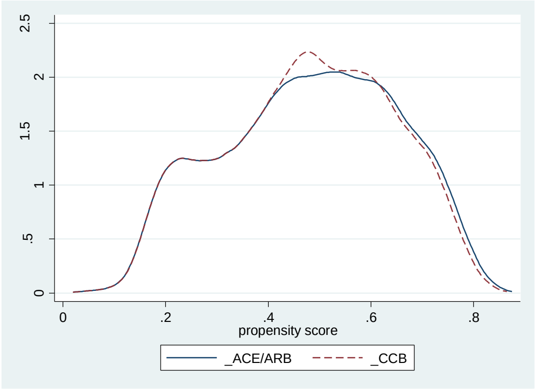
</td></tr>
<tr><td>Black</td><td>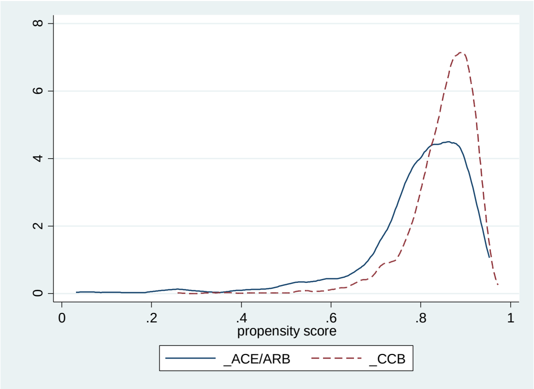
</td><td>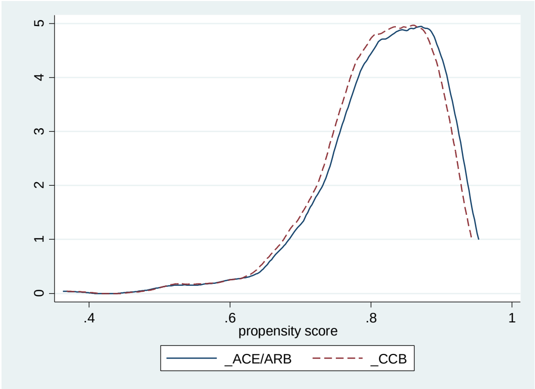
</td></tr>
</table>

Appendix 3: Descriptive characteristics and propensity score distributions before and after matching, thiazides vs CCB

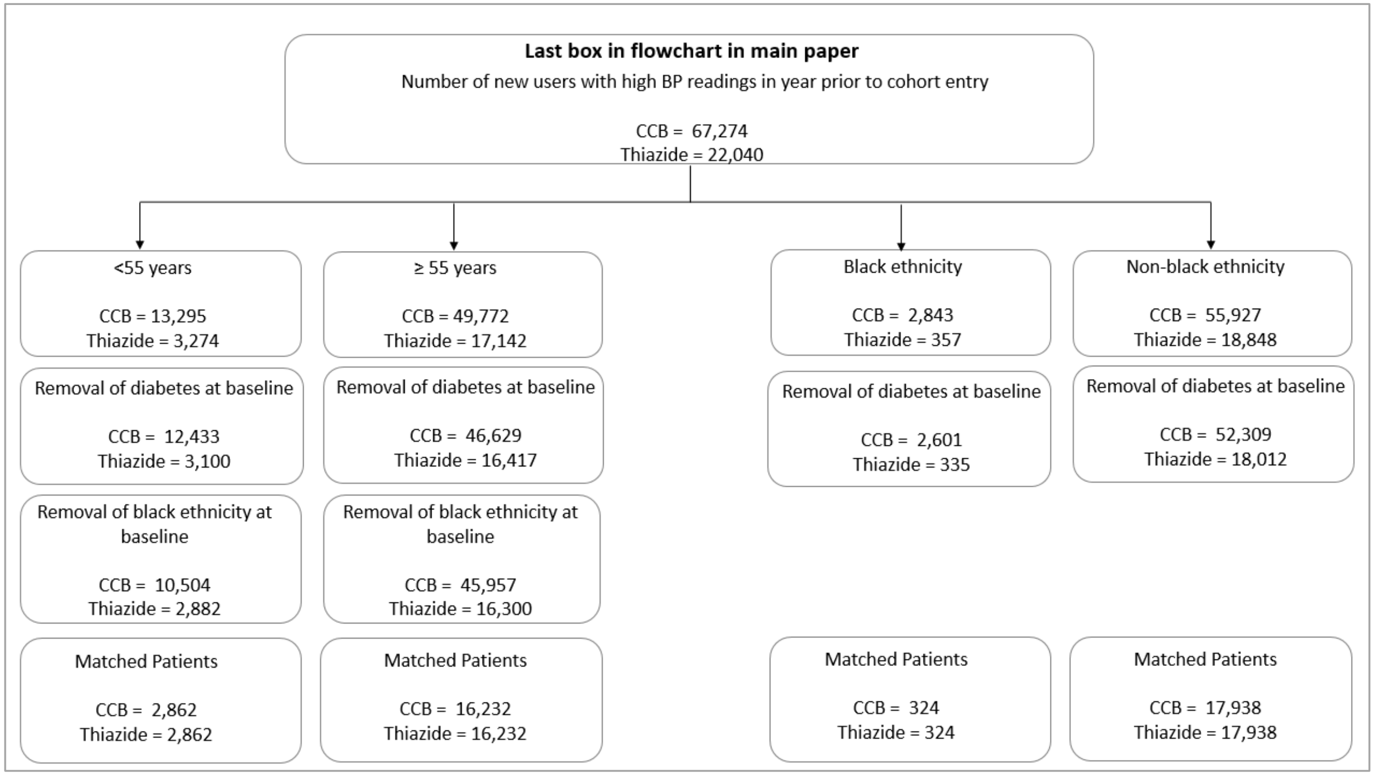

Appendix 3 Figure 1: Flowchart describing construction for each stratum for thiazide versus CCB comparison

Appendix 3 Table 1: Descriptive characteristics for thiazides *versus* calcium channel blockers (CCB) in matched stratum specific cohorts

	Aged <55yrs		Aged ≥55yrs		Non-Black		Black	
	CCB	Thiazides	CCB	Thiazides	CCB	Thiazides	CCB	Thiazides
	2862	2862	16232	16232	17938	17938	324	324
Females	1881 (65.7)	1902 (66.5)	10392 (64.0)	10414 (64.2)	11708 (65.3)	11636 (64.9)	222 (68.5)	223 (68.8)
Age (years)								
<50	1751 (61.2)	1778 (62.1)	0 (0.0)	0 (0.0)	1616 (9.0)	1616 (9.0)	161 (49.7)	161 (49.7)
50-54	1111 (38.8)	1084 (37.9)	0 (0.0)	0 (0.0)	985 (5.5)	985 (5.5)	54 (16.7)	54 (16.7)
55-59	0 (0.0)	0 (0.0)	2048 (12.6)	2048 (12.6)	1861 (10.4)	1861 (10.4)	32 (9.9)	32 (9.9)
60-64	0 (0.0)	0 (0.0)	3035 (18.7)	3035 (18.7)	2795 (15.6)	2795 (15.6)	19 (5.9)	19 (5.9)
65-69	0 (0.0)	0 (0.0)	2879 (17.7)	2879 (17.7)	2708 (15.1)	2708 (15.1)	26 (8.0)	26 (8.0)
70-74	0 (0.0)	0 (0.0)	2819 (17.4)	2819 (17.4)	2676 (14.9)	2676 (14.9)	14 (4.3)	14 (4.3)
75+	0 (0.0)	0 (0.0)	5451 (33.6)	5451 (33.6)	5297 (29.5)	5297 (29.5)	18 (5.6)	18 (5.6)
Ethnicity**								
White	2304 (80.5)	2322 (81.1)	14478 (89.2)	14689 (90.5)	16833 (93.8)	17023 (94.9)	0 (0.0)	0 (0.0)
South asian	142 (5.0)	126 (4.4)	280 (1.7)	178 (1.1)	444 (2.5)	303 (1.7)	0 (0.0)	0 (0.0)
Black	0 (0.0)	0 (0.0)	0 (0.0)	0 (0.0)	0 (0.0)	0 (0.0)	324 (100.0)	324 (100.0)
Other/mixed/unknown	187 (6.5)	149 (5.2)	459 (2.8)	463 (2.9)	661 (3.7)	612 (3.4)	0 (0.0)	0 (0.0)
Missing	229 (8.00)	265 (9.26)	1015 (6.25)	902 (5.56)	0 (0.00)	0 (0.00)	0 (0.00)	0 (0.00)
BMI**								
Underweight <18.5	16 (0.6)	14 (0.5)	319 (2.0)	302 (1.9)	309 (1.7)	308 (1.7)	~	~
Healthy weight 18.5-24.9	454 (15.9)	462 (16.1)	4983 (30.7)	5000 (30.8)	5109 (28.5)	5157 (28.7)	75 (23.1)	54 (16.7)
Overweight 25-29.9	842 (29.4)	856 (29.9)	5936 (36.6)	6034 (37.2)	6472 (36.1)	6473 (36.1)	80 (24.7)	92 (28.4)
Obesity >=30	1431 (50.0)	1449 (50.6)	4251 (26.2)	4219 (26.0)	5267 (29.4)	5300 (29.5)	160 (49.4)	170 (52.5)
Missing	119 (4.2)	81 (2.8)	743 (4.6)	677 (4.2)	781 (4.4)	700 (3.9)	7 (2.2)	7 (2.2)
SMOKING**								
Non-smoker	1074 (37.5)	1086 (37.9)	5642 (34.8)	5755 (35.5)	6255 (34.9)	6354 (35.4)	183 (56.5)	185 (57.1)
Current smoker	700 (24.5)	723 (25.3)	2548 (15.7)	2543 (15.7)	3048 (17.0)	3073 (17.1)	46 (14.2)	50 (15.4)
Ex-smoker	1085 (37.9)	1050 (36.7)	7993 (49.2)	7895 (48.6)	8575 (47.8)	8470 (47.2)	95 (29.3)	89 (27.5)
Missing	3 (0.1)	3 (0.1)	49 (0.3)	39 (0.2)	60 (0.3)	41 (0.2)	0 (0.0)	0 (0.0)
ALCOHOL**								
Non-drinker	268 (9.4)	304 (10.6)	1771 (10.9)	1863 (11.5)	2033 (11.3)	2072 (11.6)	111 (34.3)	102 (31.5)
Current drinker	2221 (77.6)	2212 (77.3)	12029 (74.1)	12090 (74.5)	13250 (73.9)	13377 (74.6)	178 (54.9)	177 (54.6)
Ex-drinker	226 (7.9)	228 (8.0)	1588 (9.8)	1551 (9.6)	1729 (9.6)	1719 (9.6)	22 (6.8)	32 (9.9)
Missing	147 (5.1)	118 (4.1)	844 (5.2)	728 (4.5)	926 (5.2)	770 (4.3)	13 (4.0)	13 (4.0)
Comorbidities								
Myocardial Infarction	~	~	121 (0.7)	130 (0.8)	133 (0.7)	130 (0.7)	~	~
Stroke	30 (1.0)	27 (0.9)	642 (4.0)	637 (3.9)	646 (3.6)	660 (3.7)	~	~
Heart Failure	~	~	83 (0.5)	90 (0.6)	104 (0.6)	92 (0.5)	~	~
Peripheral Vascular Disease	26 (0.9)	25 (0.9)	707 (4.4)	711 (4.4)	768 (4.3)	727 (4.1)	~	~
Diabetes	0 (0.0)	0 (0.0)	0 (0.0)	0 (0.0)	0 (0.0)	0 (0.0)	0 (0.0)	0 (0.0)
Depression	294 (10.3)	301 (10.5)	1027 (6.3)	986 (6.1)	1213 (6.8)	1251 (7.0)	7 (2.2)	13 (4.0)
COPD	53 (1.9)	45 (1.6)	1011 (6.2)	1013 (6.2)	1067 (5.9)	1040 (5.8)	6 (1.9)	~
Cancer	102 (3.6)	105 (3.7)	2242 (13.8)	2267 (14.0)	2365 (13.2)	2330 (13.0)	9 (2.8)	13 (4.0)
Herpes Zoster	124 (4.3)	115 (4.0)	1618 (10.0)	1595 (9.8)	1659 (9.2)	1623 (9.0)	8 (2.5)	8 (2.5)
Gout	51 (1.8)	54 (1.9)	495 (3.0)	497 (3.1)	549 (3.1)	529 (2.9)	6 (1.9)	5 (1.5)
Angioedema	~	~	~	~	~	~	~	~
CKD**								
no CKD	1586 (55.4)	1626 (56.8)	8828 (54.4)	8623 (53.1)	9731 (54.2)	9601 (53.5)	170 (52.5)	166 (51.2)
stage 3a	31 (1.1)	36 (1.3)	1653 (10.2)	1850 (11.4)	1634 (9.1)	1811 (10.1)	14 (4.3)	17 (5.2)
stage 3b	~	~	333 (2.1)	319 (2.0)	311 (1.7)	319 (1.8)	~	~
stage 4	0 (0.0)	0 (0.0)	38 (0.2)	33 (0.2)	39 (0.2)	33 (0.2)	~	~
stage 5	0 (0.0)	~	7 (0.0)	~	12 (0.1)	~	~	~
Missing	1244 (43.5)	1198 (41.9)	5373 (33.1)	5406 (33.3)	6211 (34.6)	6172 (34.4)	138 (42.6)	136 (42.0)
Drugs								
Antiplatelet agents	140 (4.9)	132 (4.6)	3001 (18.5)	2944 (18.1)	3002 (16.7)	3026 (16.9)	19 (5.9)	21 (6.5)
Statins	173 (6.0)	168 (5.9)	3296 (20.3)	3319 (20.4)	3364 (18.8)	3368 (18.8)	27 (8.3)	26 (8.0)

Proton Pump Inhibitors	846 (29.6)	845 (29.5)	6183 (38.1)	6190 (38.1)	6932 (38.6)	6838 (38.1)	83 (25.6)	79 (24.4)
Insulin	0 (0.0)	0 (0.0)	0 (0.0)	0 (0.0)	0 (0.0)	0 (0.0)	~	~
Loop diuretics	132 (4.6)	120 (4.2)	1167 (7.2)	1131 (7.0)	1257 (7.0)	1234 (6.9)	7 (2.2)	11 (3.4)
NSAIDs	1799 (62.9)	1820 (63.6)	11103 (68.4)	10872 (67.0)	12221 (68.1)	12092 (67.4)	195 (60.2)	212 (65.4)
Primary Care Consultations**								
<5 consultations	779 (27.2)	799 (27.9)	4069 (25.1)	4068 (25.1)	4357 (24.3)	4380 (24.4)	113 (34.9)	103 (31.8)
5-9 consultations	1008 (35.2)	1003 (35.0)	5494 (33.8)	5438 (33.5)	6064 (33.8)	6037 (33.7)	95 (29.3)	96 (29.6)
10-14 consultations	477 (16.7)	462 (16.1)	3000 (18.5)	3076 (19.0)	3414 (19.0)	3404 (19.0)	61 (18.8)	62 (19.1)
15-19 consultations	239 (8.4)	215 (7.5)	1577 (9.7)	1563 (9.6)	1729 (9.6)	1741 (9.7)	17 (5.2)	20 (6.2)
>=20 consultations	359 (12.5)	383 (13.4)	2092 (12.9)	2087 (12.9)	2374 (13.2)	2376 (13.2)	38 (11.7)	43 (13.3)
Missing	0 (0.0)	0 (0.0)	0 (0.0)	0 (0.0)	0 (0.0)	0 (0.0)	0 (0.0)	0 (0.0)
Systolic BP	156.9 (17.3)	157.4 (16.4)	166.7 (17.5)	166.7 (16.7)	165.3 (17.7)	165.2 (16.9)	158.5 (17.4)	159.6 (16.2)
Diastolic BP	99.0 (10.6)	97.5 (9.8)	91.0 (11.6)	90.8 (11.0)	92.2 (11.8)	91.6 (11.1)	96.9 (10.2)	97.7 (10.1)

<table>
<tr><td></td><td>Before Matching</td><td>After Matching</td></tr>
<tr><td><55years</td><td>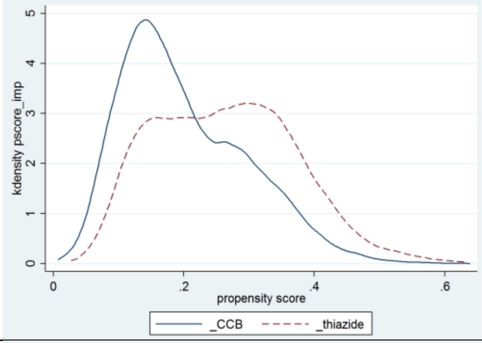</td><td>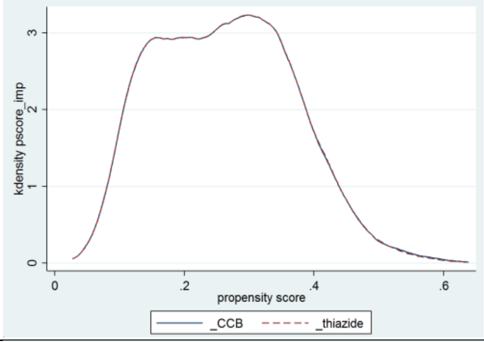</td></tr>
<tr><td>≥55years</td><td>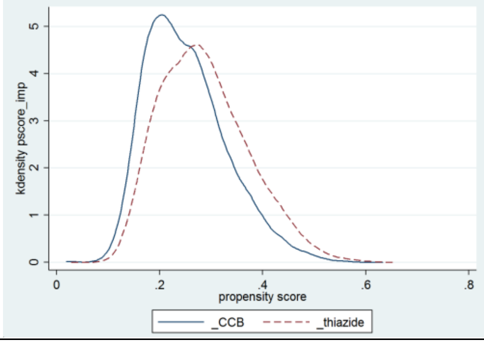</td><td>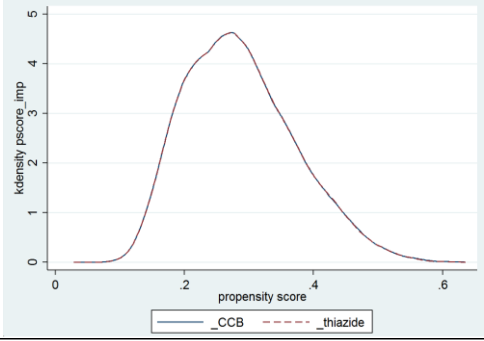</td></tr>
<tr><td></td><td></td><td></td></tr>
<tr><td>Non-Black</td><td>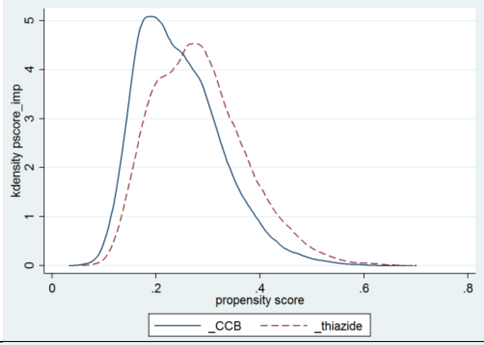</td><td>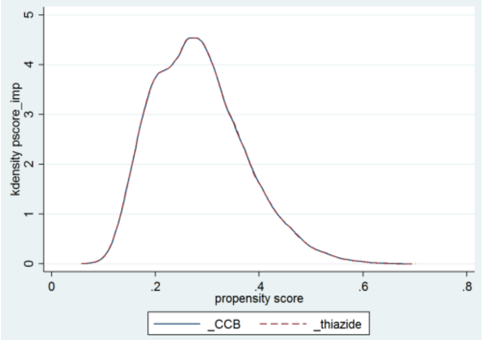</td></tr>
<tr><td>Black</td><td>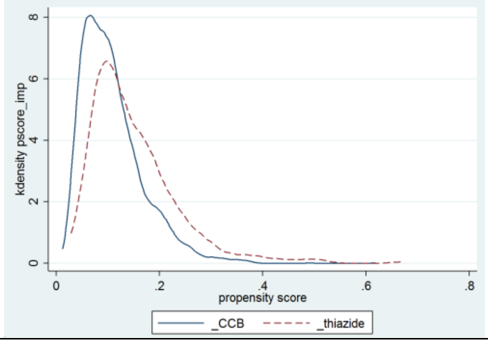</td><td>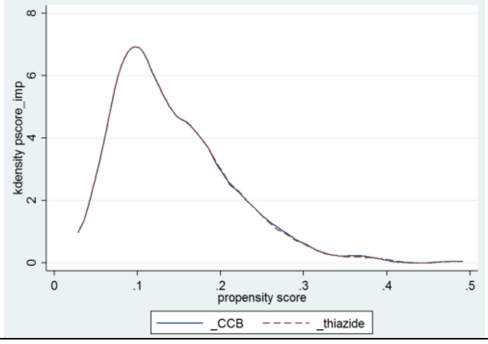</td></tr>
</table>

Appendix 4: Diastolic blood pressure results

Appendix 4 Table 1: Difference in diastolic blood pressure since index date for CCB *versus* ACE-I/ARB and thiazide *versus* CCB.

		**Difference in Diastolic BP between CCB and ACE-I/ARB (99% CI)**		
	**n**	**12 weeks**	**26 weeks**	**52 weeks**
Age <55*	20,964	-0.98 (-1.39, -0.56)	-0.28 (-0.79, 0.22)	0.11 (-0.76, 0.97)
Age>55*	58,396	-1.06 (-1.39, -0.72)	-0.14 (-0.47, 0.18)	0.01 (-0.47, 0.49)
Non-black**	73,726	-1.06 (-1.27, -0.85)	-0.20 (-0.43, 0.02)	-0.02 (-0.44, 0.40)
Black**	894	-2.46 (-4.11, -0.82)	-0.96 (-3.40, 1.49)	0.99 (-2.48, 4.46)
		**Difference in Diastolic BP between thiazide and CCB (99% CI)**		
Age <55*	5,724	1.29 (0.40, 2.18)	0.62 (-0.22, 1.45)	0.83 (-0.75, 2.42)
Age>55*	32,464	1.80 (1.44, 2.15)	1.36 (1.00, 1.71)	0.80 (0.15, 1.45)
Non-black**	35,876	1.75 (1.41, 2.08)	1.24 (0.93, 1.56)	0.84 (0.29, 1.40)
Black**	648	0.82 (-1.11, 2.74)	-0.26 (-2.86, 2.34)	1.92 (-3.04, 6.89)

*In a non-black, non-diabetic population

** In a non-diabetic population

All analyses are 1:1 PS matched within each stratum. Each n consists of half CCB and half ACEI/ARB (or half thiazide and half CCB).

For the CCB vs ACEI/ARB comparison a negative result means CCB produced larger reductions in systolic BP and a positive results indicates that ACEI/ARB produced larger reductions in systolic BP. For the thiazide vs CCB comparison a negative result means thiazides produced larger reductions in systolic BP and a positive results indicates that CCB produced larger reductions in systolic BP.

Appendix 5: Diagrams of diastolic blood pressure results

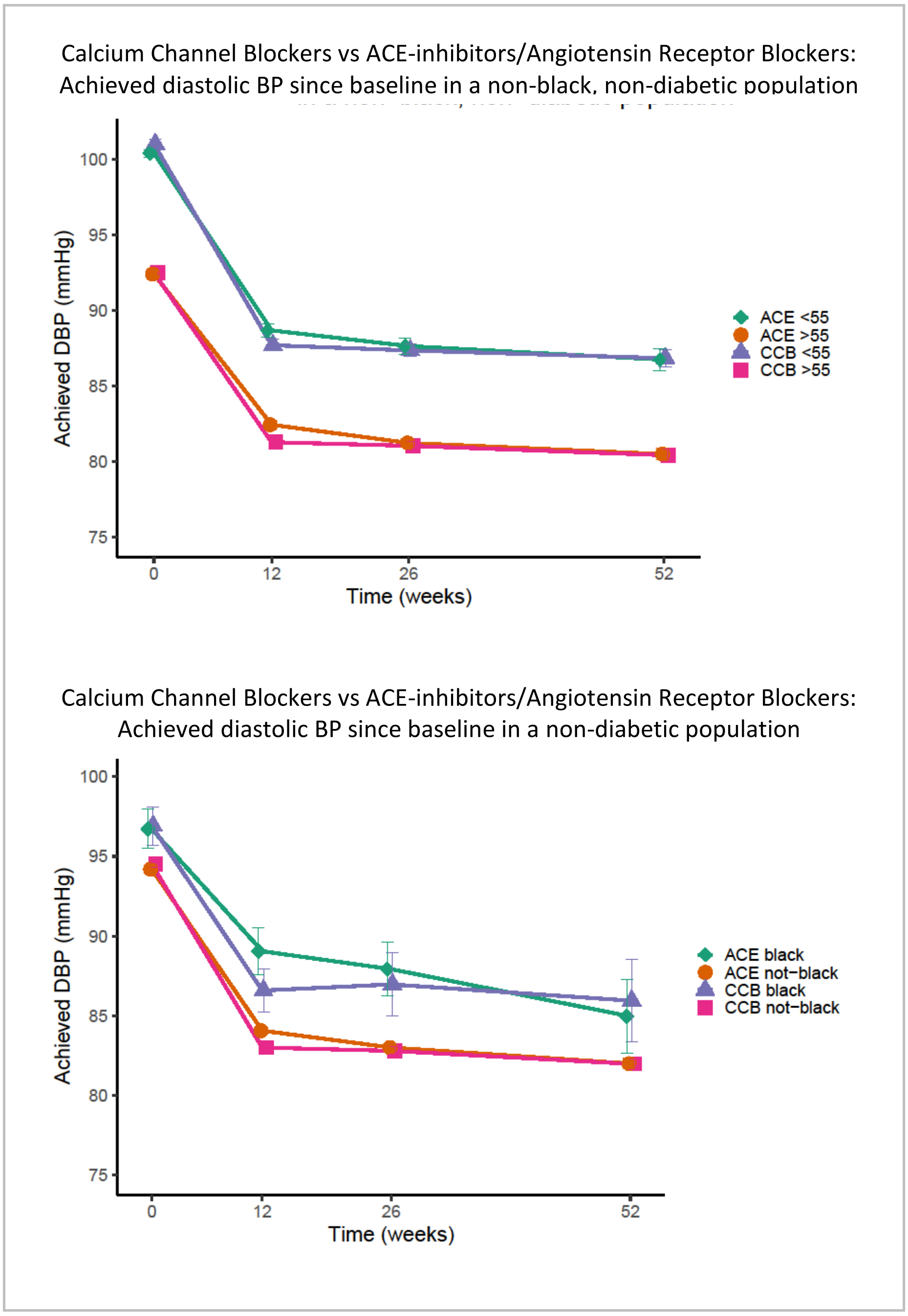

Appendix 5 Figure 1: Achieved diastolic blood pressures for calcium channel blocker (CCB) and ACE-inhibitor/angiotensin receptor blocker (ACE-I/ARB) in each stratum

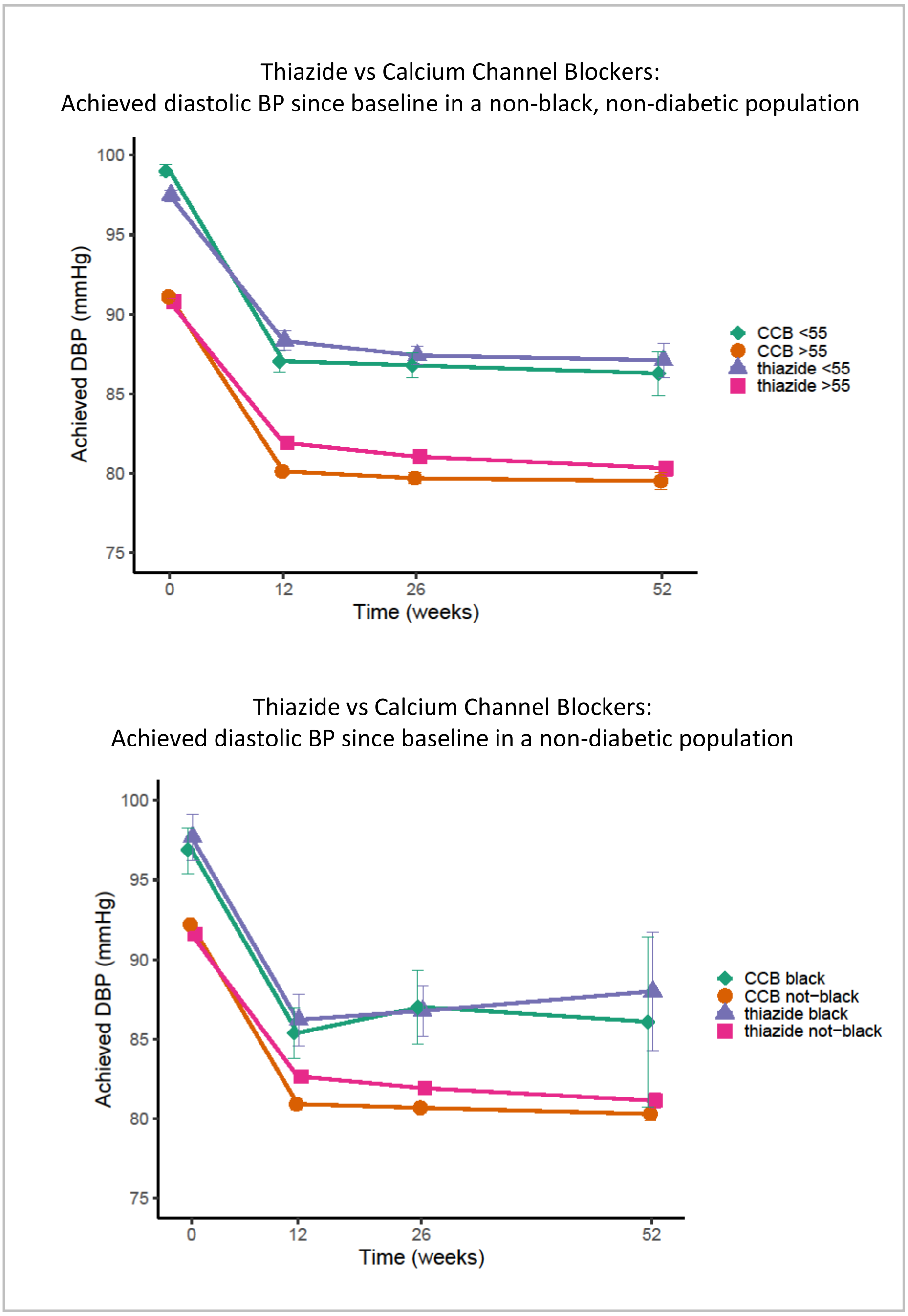

Appendix 5 Figure 2: Achieved diastolic blood pressures for thiazide and calcium channel blocker (CCB) in each stratum

Appendix 6: 95% CI for changes in systolic blood pressure

Appendix 6 Table 1: Difference in systolic blood pressure since drug initiation for calcium channel blockers (CCB) *versus* ACE-inhibitors/angiotensin receptor blockers (ACEI/ARB) and thiazide *versus* CCB, 95% CI.

		**Difference between CCB and ACEI/ARB for change in systolic BP(mmHg) since baseline (95% CI)**		
	**n**	**12 weeks**	**26 weeks**	**52 weeks**
Age <55*	20,964	-1.69 (-2.32, -1.06)	-0.48 (-1.24, 0.27)	-0.53 (-1.62, 0.57)
Age>55*	58,396	-0.40 (-0.84, 0.04)	0.63 (0.20, 1.07)	0.85 (0.12, 1.58)
Non-black**	73,726	-0.98 (-1.37, -0.59)	0.11 (-0.30, 0.51)	0.08 (-0.60, 0.75)
Black**	894	-2.15 (-5.21, 0.91)	0.55 (-2.45, 3.55)	2.28 (-3.53, 8.09)
		**Difference between thiazide and CCB for change in systolic BP(mmHg) since baseline (95% CI)**		
Age <55*	5,724	1.51 (0.34, 2.69)	0.07 (-1.28, 1.41)	0.34 (-1.74, 2.42)
Age>55*	32,464	2.16 (1.55, 2.77)	1.43 (0.77, 2.10)	0.17 (-0.68, 1.03)
Non-black**	35,876	2.10 (1.55, 2.65)	1.19 (0.58, 1.80)	0.32 (-0.49, 1.13)
Black**	648	-0.28 (-4.40, 3.84)	-1.75 (-5.73, 2.23)	2.01 (-3.68, 7.69)

*In a non-black, non-diabetic population
** In a non-diabetic population
All analyses are 1:1 PS matched within each stratum. Each n consists of half CCB and half ACEI/ARB (or half thiazide and half CCB).
For the CCB vs ACEI/ARB comparison a negative result means CCB produced larger reductions in systolic BP and a positive results indicates that ACEI/ARB produced larger reductions in systolic BP. For the thiazide vs CCB comparison a negative result means thiazides produced larger reductions in systolic BP and a positive results indicates that CCB produced larger reductions in systolic BP.

Appendix 7: Negative and positive outcomes

Appendix 7 Table 1: Time dependent hazard ratios in time to event analyses for negative outcomes Ankle Swelling and Gout in the whole matched population

	CCB vs ACEI/ARB<br>HR (99% CI)	Thiazide vs CCB<br>HR (99% CI)
**Ankle Swelling**		
0-3months	6.77 (4.87 – 9.41)	0.30 (0.22 – 0.41)
3-6months	3.43 (2.55 – 4.61)	0.42 (0.30 – 0.60)
6-9 months	2.02 (1.48 – 2.75)	0.59 (0.39 – 0.89)
9+months	1.34 (1.23 – 1.47)	0.85 (0.75 – 0.96)
**Gout**		
0-3months	1.22 (0.74 – 2.04)	1.20 (0.53 – 2.68)
3-6months	0.89 (0.51 – 1.57)	1.82 (0.59 – 5.62)
6-9 months	1.15 (0.62 – 2.15)	2.20 (0.77 – 6.33)
9months+	0.89 (0.77 – 1.02)	1.17 (0.97 – 1.40)

Note: We have presented the time stratified hazard ratios for risk of Gout and Ankle Swelling in both drug comparisons above. We opted to present the hazard ratio averaged over time in the main manuscript because it succinctly and clearly communicates the main point of these analyses which was to demonstrate that our data and methods could generate expected drug-outcome associations.

Analysis	CCB Person Yrs	CCB Outcomes	ACEI/ARB Person Yrs	ACEI/ARB Outcomes	HR	99% CI
Negative Control Outcome: Zoster						
<55years	40324.1	145	42958.7	169	0.95	0.67, 1.35
>=55years	104451.0	745	121041.8	982	0.90	0.79, 1.02
Non−black	134184.0	851	152401.4	1083	0.90	0.80, 1.02
Black	1624.6	7	1872.8	~	2.77	0.33, 23.01
Positive Control Outcome: Angioedema						
<55years	41977.5	17	44714.8	15	1.08	0.34, 3.40
>=55years	115634.5	34	134305.6	59	0.72	0.40, 1.30
Non−black	145918.7	52	166448.9	77	0.79	0.48, 1.28
Black	1664.7	~	1963.2	~	3.93	0.18, 87.65
Positive Control Outcome: Gout						
<55years	38812.1	204	42313.6	221	0.96	0.75, 1.24
>=55years	107095.1	561	124589.6	722	0.89	0.76, 1.04
Non−black	136973.3	706	155552.8	929	0.90	0.78, 1.03
Black	1566.5	10	1915.5	~	3.27	0.49, 21.82
Positive Control Outcome: Ankle Swelling						
<55years	39700.6	459	43344.2	258	1.82	1.44, 2.31
>=55years	104492.4	2234	125619.3	1441	1.83	1.67, 2.01
Non−black	133808.8	2609	157021.3	1651	1.81	1.61, 2.03
Black	1561.6	22	1833.3	15	1.77	0.74, 2.06

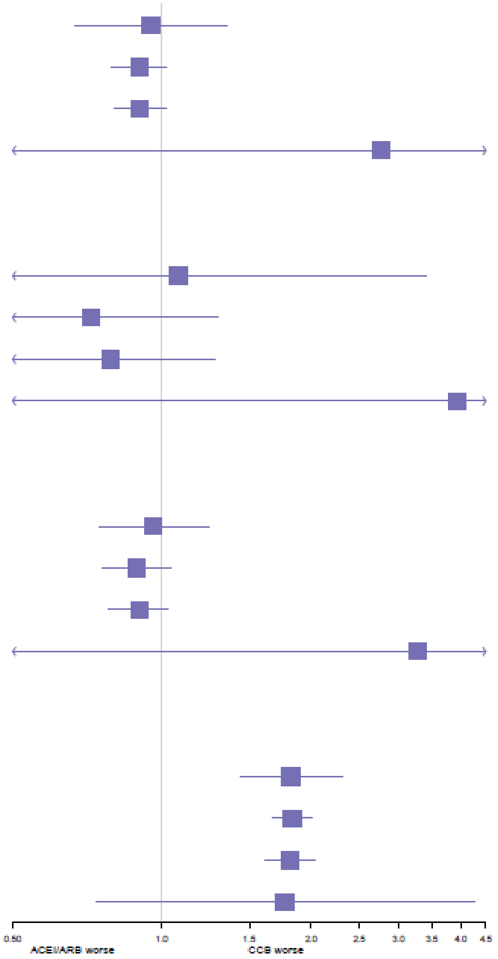

Appendix 7 Figure 1: Negative and positive outcomes for calcium channel blockers (CCB) versus ACE-inhibitors/angiotensin receptor blockers (ACEI/ARB) in age-based and ethnicity-based strata.

~ is used when cell sizes are <5

Analysis	Thiazide Person Yrs	Thiazide Outcomes	CCB Person Yrs	CCB Outcomes	HR	99% CI
Negative Control Outcome: Zoster						
<55years	13801.8	63	11272.7	38	1.28	0.76, 2.16
>=55years	68407.9	636	56087.5	438	1.16	0.96, 1.39
Non−black	77115.0	671	63174.3	487	1.16	0.96, 1.41
Black	1523.8	0	1137.8	~	na	na,na
Positive Control Outcome: Angioedema						
<55years	14554.7	5	11647.0	~	1.75	0.12, 26.63
>=55years	77452.7	20	63321.1	22	0.83	0.32, 2.15
Non−black	86448.0	25	79671.7	19	0.91	0.34,2.47
Black	1552.1	0	1150.6	~	na	na, na
Positive Control Outcome: Gout						
<55years	14133.6	64	11317.2	47	1.19	0.56, 2.54
>=55years	73990.4	452	60878.7	264	1.38	1.09, 1.75
Non−black	82729.2	493	67854.3	299	1.41	1.14,1.75
Black	1537.0	6	1289.5	~	1.22	0.12, 12.23
Positive Control Outcome: Ankle Swelling						
<55years	13391.4	114	10917.7	132	0.73	0.50, 1.08
>=55years	69189.0	1207	56328.2	1467	0.70	0.62, 0.78
Non−black	77286.8	1281	63248.2	1562	0.69	0.62, 0.77
Black	1463.4	14	1114.2	17	0.60	0.20, 1.76

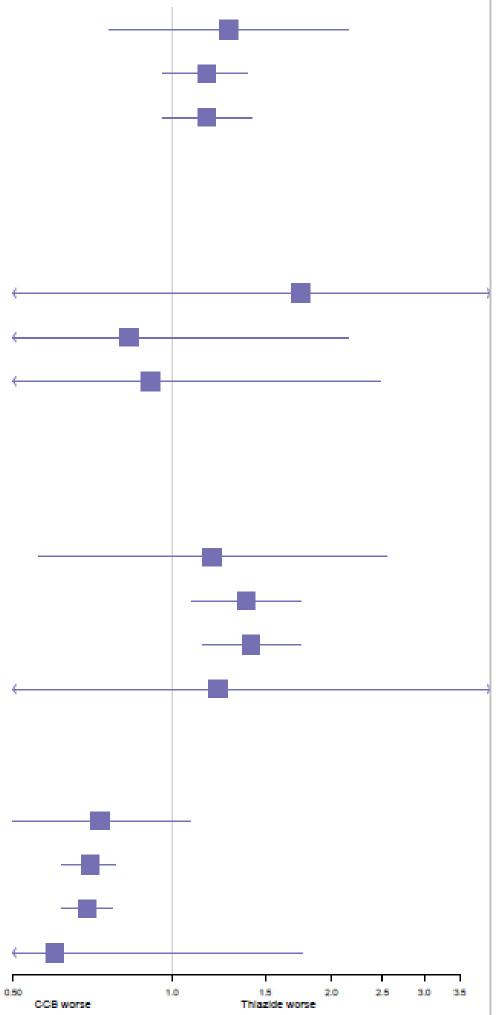

Appendix 7 Figure 2: Negative and positive outcomes for thiazide versus calcium channel blockers (CCB) in age-based and ethnicity-based strata.
~ is used when cell sizes are <5

Appendix 8: Comparison of thiazide-like diuretics with calcium channel blockers

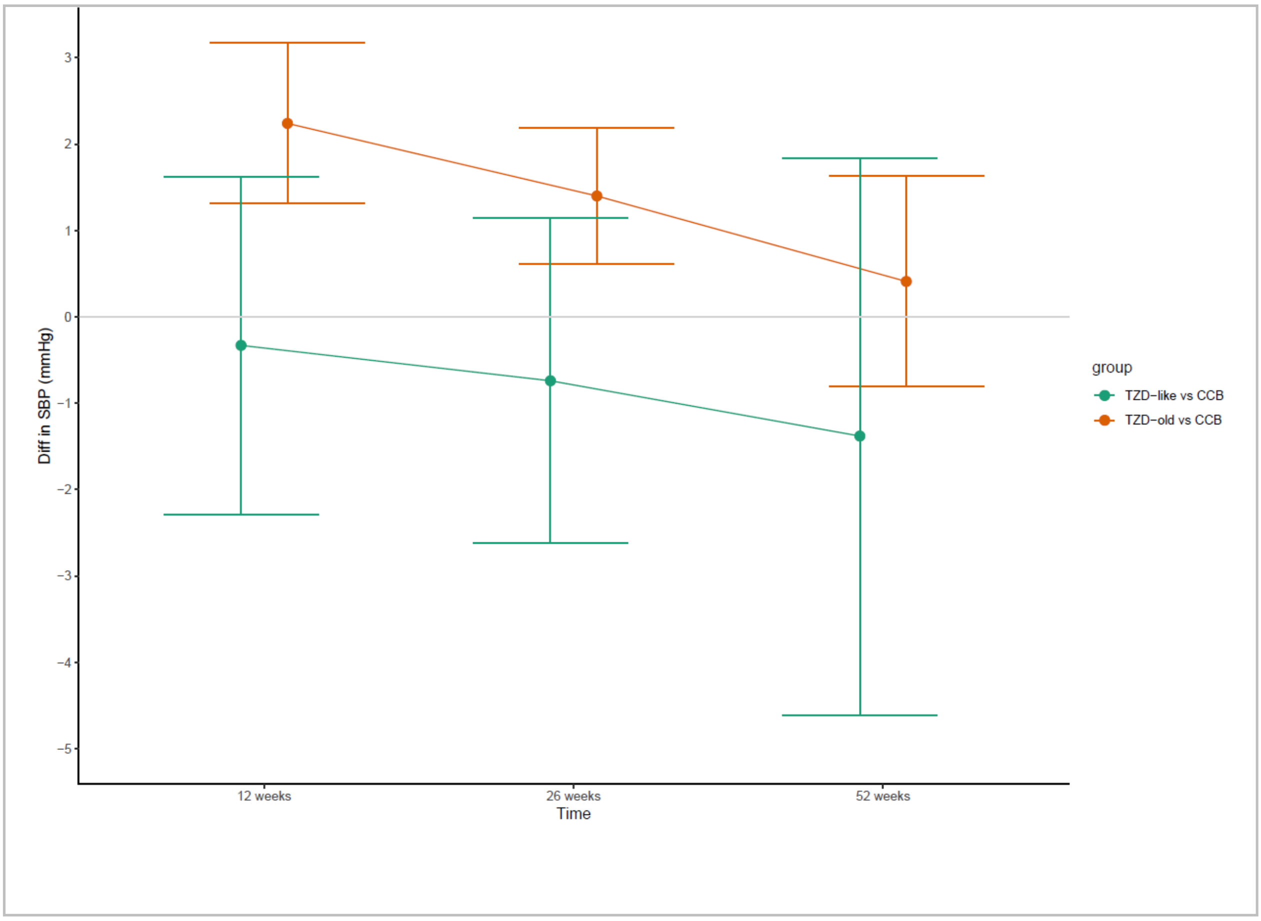

Appendix 8 Figure 1: Comparison of thiazide-like diuretics (chlortalidone and indapamide) with calcium channel blockers (CCB). Comparison of traditional thiazide diuretics (eg bendroflumethazide) with CCB.

Data-points below the null line indicate that thiazides are better at reducing BP. Data-points above the null line indicate that CCB are better at reducing BP.

Appendix 9: On treatment analysis

Appendix 9 Table 1: Difference in systolic blood pressure since index date for CCB *versus* ACE-I/ARB and thiazide *versus* CCB in an on-treatment analysis

		**Difference between CCB and ACEI/ARB for change in systolic BP(mmHg) since baseline (99% CI)**		
	**n**	**12 weeks**	**26 weeks**	**52 weeks**
Age <55*	19,228	0.22 (-0.66, 1.11)	0.35 (-0.83, 1.54)	1.01 (-0.90, 2.93)
Age>55*	54,149	-0.19 (-0.95, 0.57)	0.17 (-0.51, 0.84)	-0.09 (-1.21, 1.02)
Non-black**	35,876	-0.35 (-0.94, 0.24)	0.03 (-0.61, 0.67)	-0.30 (-1.28, 0.67)
Black**	648	-3.65 (-7.99, 0.70)	-5.16 (-11.13, 0.81)	-3.36 (-12.10, 5.37)
		**Difference between thiazide and CCB for change in systolic BP(mmHg) since baseline (99% CI)**		
Age <55*	5,056	3.04 (1.17, 4.90)	3.06 (0.72, 5.40)	0.40 (-3.03, 3.83)
Age>55*	29,965	4.14 (3.23, 5.04)	4.12 (3.05, 5.20),	3.91 (2.39, 5.44)
Non-black**	32,833	4.05 (3.21, 4.88)	3.93 (2.89, 4.97)	3.55 (2.03, 5.07)
Black**	555	2.73 (-3.51, 8.98)	1.46 (-4.95, 7.86)	3.47 (-8.51, 15.46)

*In a non-black, non-diabetic population

** In a non-diabetic population

All analyses are 1:1 PS matched within each stratum. Each n consists of half CCB and half ACE-i/ARB (or half thiazide and half CCB).

For the CCB vs ACE-I/ARB comparison a negative result means CCB produced larger reductions in systolic BP and a positive results indicates that ACEI/ARB produced larger reductions in systolic BP. For the thiazide vs CCB comparison a negative result means thiazides produced larger reductions in systolic BP and a positive results indicates that CCB produced larger reductions in systolic BP. There was a median of 4 (IQR 2-6) BP measurements available during one year follow up for each patient.

On treatment analysis: patients follow-up BP data were censored if the patient switched to another drug class, added a drug from another class or discontinued their anti-hypertensive treatment (drug classes: thiazides, CCB, ACE-I/ARB, beta-blockers, alpha-blockers, potassium sparing diuretics, aldosterone antagonists and other).

Of 87,440 ACEI/ARB initiators, n= 41,940 had a treatment switch, addition or discontinuation. Median time to regimen change was 189 days (IQR 51 – 691)

Of 67,274 CCB initiators, n= 35,274 had a treatment switch, addition or discontinuation. Median time to regimen change was 100 days (IQR 34 – 416)

Of 22,040 thiazide initiators, n= 14,622 had a treatment switch, addition or discontinuation. Median time to regimen change was 86 days (IQR 29 – 430)

Appendix 10: Complete case analysis

Appendix 10 Table 1: Complete case analysis

	n	12 weeks	26 weeks	52 weeks
		**Difference between CCB and ACEI/ARB for change in systolic BP(mmHg) since baseline (99% CI)**		
Age <55*	11,660	-1.77 (-2.77, -0.77)	-0.45 (-1.63, 0.73)	-0.84 (-2.40, 0.72)
Age>55*	40,890	-0.46 (-1.06, 0.14)	0.52 (-0.09, 1.12)	1.01 (-0.002, 2.02)
Non-black**	49,186	-1.06 (-1.61, -0.52)	-0.12 (-0.69, 0.45)	-0.04 (-0.96, 0.89)
Black**	592	-2.21 (-6.58, 2.17)	0.27 (-4.21, 4.76)	0.000 (-6.78, 6.78)
		**Difference between thiazide and CCB for change in systolic BP(mmHg) since baseline (99% CI)**		
Age <55*	3,472	1.22 (-0.59, 3.03)	0.04 (-1.84, 1.92)	-1.01 (-3.87, 1.85)
Age>55*	21,448	1.84 (0.98, 2.69)	0.83 (0.04, 1.63)	-0.04 (-1.36, 1.28)
Non-black**	23,550	1.84 (1.01, 2.67)	0.89 (0.07, 1.70)	-0.01 (-1.26, 1.25)
Black**	372	-1.11 (-7.29, 5.07)	0.88 (-5.00, 6.77)	2.75 (-6.01, 11.51)

*In a non-black, non-diabetic population

** In a non-diabetic population

All analyses are 1:1 PS matched within each stratum. Each n consists of half CCB and half ACEI/ARB (or half thiazide and half CCB).

For the CCB vs ACEI/ARB comparison a negative result means CCB produced larger reductions in systolic BP and a positive results indicates that ACE-I/ARB produced larger reductions in systolic BP. For the thiazide vs CCB comparison a negative result means thiazides produced larger reductions in systolic BP and a positive results indicates that CCB produced larger reductions in systolic BP. There was a median of 4 (IQR 2-6) BP measurements available during one year follow up for each patient.

Appendix 11: RECORD-PE checklist

The RECORD statement for pharmacoepidemiology (RECORD-PE) checklist of items, extended from the STROBE and RECORD statements, which should be reported in non-interventional pharmacoepidemiological studies using routinely collected health data

Item No	STROBE items	RECORD items	RECORD-PE items	Page No
**Title and abstract**				
1	(a) Indicate the study's design with a commonly used term in the title or the abstract.<br>(b) Provide in the abstract an informative and balanced summary of what was done and what was found.	1.1: The type of data used should be specified in the title or abstract. When possible, the name of the databases used should be included.<br>1.2: If applicable, the geographical region and timeframe within which the study took place should be reported in the title or abstract.<br>1.3: If linkage between databases was conducted for the study, this should be clearly stated in the title or abstract.	—	1 & 2
**Introduction**				
Background rationale				
2	Explain the scientific background and rationale for the investigation being reported.	—	—	5
Objectives				
3	State specific objectives, including any prespecified hypotheses.	—	—	5
**Methods**				
Study design				
4	Present key elements of study design early in the paper.	—	4.a: Include details of the specific study design (and its features) and report the use of multiple designs if used.<br>4.b: The use of a diagram(s) is recommended to illustrate key aspects of the study design(s), including	6

			exposure, washout, lag and observation periods, and covariate definitions as relevant.	
Setting				
5	Describe the setting, locations, and relevant dates, including periods of recruitment, exposure, follow-up, and data collection.	—	—	6
Participants				
6	(a) Cohort study—give the eligibility criteria, and the sources and methods of selection of participants. Describe methods of follow-up. Case-control study—give the eligibility criteria, and the sources and methods of case ascertainment and control selection. Give the rationale for the choice of cases and controls. Cross sectional study—give the eligibility criteria, and the sources and methods of selection of participants.<br>(b) Cohort study—for matched studies, give matching criteria and number of exposed and unexposed. Case-control study—for matched studies, give matching criteria and the number of controls per case.	6.1: The methods of study population selection (such as codes or algorithms used to identify participants) should be listed in detail. If this is not possible, an explanation should be provided.<br>6.2: Any validation studies of the codes or algorithms used to select the population should be referenced. If validation was conducted for this study and not published elsewhere, detailed methods and results should be provided.<br>6.3: If the study involved linkage of databases, consider use of a flow diagram or other graphical display to demonstrate the data linkage process, including the number of individuals with linked data at each stage.	6.1.a: Describe the study entry criteria and the order in which these criteria were applied to identify the study population. Specify whether only users with a specific indication were included and whether patients were allowed to enter the study population once or if multiple entries were permitted. See explanatory document for guidance related to matched designs.	6
Variables				
7	Clearly define all outcomes, exposures, predictors, potential confounders, and effect modifiers. Give diagnostic criteria, if applicable.	7.1: A complete list of codes and algorithms used to classify exposures, outcomes, confounders, and effect modifiers should be provided. If these cannot be reported, an explanation should be provided.	7.1.a: Describe how the drug exposure definition was developed.<br>7.1.b: Specify the data sources from which drug exposure information for individuals was obtained.<br>7.1.c: Describe the time window(s) during which an individual is considered exposed to the drug(s). The rationale for selecting a particular	6 & 7

			time window should be provided. The extent of potential left truncation or left censoring should be specified.<br>7.1.d: Justify how events are attributed to current, prior, ever, or cumulative drug exposure.<br>7.1.e: When examining drug dose and risk attribution, describe how current, historical or time on therapy are considered.<br>7.1.f: Use of any comparator groups should be outlined and justified.<br>7.1.g: Outline the approach used to handle individuals with more than one relevant drug exposure during the study period.	
Data sources/measurement				
8	For each variable of interest, give sources of data and details of methods of assessment (measurement). Describe comparability of assessment methods if there is more than one group.	—	8.a: Describe the healthcare system and mechanisms for generating the drug exposure records. Specify the care setting in which the drug(s) of interest was prescribed.	6
Bias				
9	Describe any efforts to address potential sources of bias.	—	—	6-8
Study size				
10	Explain how the study size was arrived at.	—	—	flowchart
Quantitative variables				
11	Explain how quantitative variables were handled in the analyses. If applicable, describe which groupings were chosen, and why.	—	—	7
Statistical methods				
12	(a) Describe all statistical methods, including those used to control for confounding.	—	12.1.a: Describe the methods used to evaluate whether the assumptions have been met.	7 & 8

	(b) Describe any methods used to examine subgroups and interactions.<br>(c) Explain how missing data were addressed.<br>(d) Cohort study—if applicable, explain how loss to follow-up was addressed. Case-control study—if applicable, explain how matching of cases and controls was addressed. Cross sectional study—if applicable, describe analytical methods taking account of sampling strategy.<br>(e) Describe any sensitivity analyses.		12.1.b: Describe and justify the use of multiple designs, design features, or analytical approaches.	
Data access and cleaning methods				
12	—	12.1: Authors should describe the extent to which the investigators had access to the database population used to create the study population.<br>12.2: Authors should provide information on the data cleaning methods used in the study.	—	7
Linkage				
12	—	12.3: State whether the study included person level, institutional level, or other data linkage across two or more databases. The methods of linkage and methods of linkage quality evaluation should be provided.	—	6
**Results**				
Participants				
13	(a) Report the numbers of individuals at each stage of the study (eg, numbers potentially eligible, examined for eligibility, confirmed eligible, included in the study, completing follow-up, and analysed).<br>(b) Give reasons for non-participation at each stage.	13.1: Describe in detail the selection of the individuals included in the study (that is, study population selection) including filtering based on data quality, data availability, and linkage. The selection of included individuals can be described in the	—	See flowchart

	(c) Consider use of a flow diagram.	text or by means of the study flow diagram.		
Descriptive data				
14	(a) Give characteristics of study participants (eg, demographic, clinical, social) and information on exposures and potential confounders.<br>(b) Indicate the number of participants with missing data for each variable of interest.<br>(c) Cohort study—summarise follow-up time (eg, average and total amount).	—	—	See table 1
Outcome data				
15	Cohort study—report numbers of outcome events or summary measures over time. Case-control study—report numbers in each exposure category, or summary measures of exposure. Cross sectional study—report numbers of outcome events or summary measures.	—	—	See table 2 (outcome was BP – a continuous outcome)
Main results				
16	(a) Give unadjusted estimates and, if applicable, confounder adjusted estimates and their precision (eg, 95% confidence intervals). Make clear which confounders were adjusted for and why they were included.<br>(b) Report category boundaries when continuous variables are categorised.<br>(c) If relevant, consider translating estimates of relative risk into absolute risk for a meaningful time period.	—	—	See Table 2
Other analyses				
17	Report other analyses done—eg, analyses of subgroups and interactions, and sensitivity analyses.	—	—	12

**Discussion**				
Key results				
18	Summarise key results with reference to study objectives.	—	—	13
Limitations				
19	Discuss limitations of the study, taking into account sources of potential bias or imprecision. Discuss both direction and magnitude of any potential bias.	19.1: Discuss the implications of using data that were not created or collected to answer the specific research question(s). Include discussion of misclassification bias, unmeasured confounding, missing data, and changing eligibility over time, as they pertain to the study being reported.	19.1.a: Describe the degree to which the chosen database(s) adequately captures the drug exposure(s) of interest.	13 & 14
Interpretation				
20	Give a cautious overall interpretation of results considering objectives, limitations, multiplicity of analyses, results from similar studies, and other relevant evidence.	—	20.a: Discuss the potential for confounding by indication, contraindication or disease severity or selection bias (healthy adherer/sick stopper) as alternative explanations for the study findings when relevant.	13
Generalisability				
21	Discuss the generalisability (external validity) of the study results.	—	—	15
**Other information**				
Funding				
22	Give the source of funding and the role of the funders for the present study and, if applicable, for the original study on which the present article is based.	—	—	16
Accessibility of protocol, raw data, and programming code				
22	—	22.1: Authors should provide information on how to access any supplemental information such as the study protocol, raw data, or programming code.	—	9

RECORD=reporting of studies conducted using observational routinely collected data; RECORD-PE=RECORD for pharmacoepidemiological research; STROBE=strengthening the reporting of observational studies in epidemiology.

*REFERENCE: Langan SM, Schmidt S, Wing K, Ehrenstein V, Nicholls S, Filion K, Klungel O, Petersen I, Sorensen H, Guttmann A, Harron K, Hemkens L, Moher D, Schneeweiss S, Smeeth L, Sturkenboom M, von Elm E, Wang S, Benchimol EI. The REporting of studies Conducted using Observational Routinely-collected health Data (RECORD) Statement for Pharmacoepidemiology (RECORD-PE). *BMJ* 2018; 363: k3532.
